# Bioactive Metabolites from the Deep-Sea-Derived Fungus *Diaporthe longicolla* FS429

**DOI:** 10.3390/md18080381

**Published:** 2020-07-23

**Authors:** Zhaoming Liu, Yuchan Chen, Saini Li, Qinglin Wang, Caiyun Hu, Hongxin Liu, Weimin Zhang

**Affiliations:** 1 State Key Laboratory of Applied Microbiology Southern China, Guangdong Provincial Key Laboratory of Microbial Culture Collection and Application, Guangdong Open Laboratory of Applied Microbiology, Guangdong Institute of Microbiology, Guangdong Academy of Sciences, 100 Central Xianlie Road, Yuexiu District, Guangzhou 510070, China; liuzm@gdim.cn (Z.L.); chenyc@gdim.cn (Y.C.); maibao66@126.com (S.L.); hucy888666@163.com (C.H.); 2 School of Life Sciences and Biomedical Center, Sun Yat-Sen University, Guangzhou 510275, China; wangqlin5@mail2.sysu.edu.cn

**Keywords:** bioactive metabolites, deep-sea derived-fungus, *Diaporthe longicolla*, bioactivities

## Abstract

The chemical investigation of a methanol extract of the deep-sea-derived fungus *Diaporthe longicolla* FS429 led to the isolation of two novel diterpenoids longidiacids A and B (**1** and **2**), two new polyketides (**3** and **4**), two new cytochalasin analogues longichalasins A and B (**6** and **8**) and three known analogues **5**, **7**, **9**. Their structures were elucidated through comprehensive spectroscopic analysis, while the absolute configurations were established by the comparison of the experimental and quantum chemical calculated ECD spectra. The structure of compound **7** was confirmed through X-ray diffraction for the first time. In the bioassays compound **8** exhibited antiproliferative effects against SF-268, with an IC_50_ value of 16.44 μM. Moreover, compounds **1** and **8** were detected to inhibit 35.4% and 53.5% of enzyme activity of *Mycobacterium tuberculosis* protein tyrosine phosphatase B (*M*ptpB) at a concentration of 50 μM.

## 1. Introduction

It is well recognized that deep-sea-derived fungi, which are collected from sediments or water at a depth over 1000 m below the surface, exhibit a rich species diversity even though they live under extreme conditions such as lack of sunlight irradiation, low temperature or oligotrophy [1,2]. The first two strains of deep-sea-derived fungi, which were identified as *Aureobasidium pullulans* and *Dendryphiella arenaria*, were isolated from the Atlantic Ocean at a depth of 4450 m by Roth et al. in 1964 [3]. After that, there was no further research about the chemical investigation of deep-sea-derived fungi until 1996, when Cui and his co-worker isolated two novel diketopiperazine derivatives spirotryprostatins A and B from *Aspergillus funigatus* [4]. Since then, the deep-sea-derived fungi have attracted more and more attention due to their abundant secondary metabolites [2], for example, 71 new aphidicolins were recently isolated from the deep-sea-derived *Botryotinia fuckeliana* [5]. Moreover, according to literature surveys, approximately 80% of the compounds exhibited potential bioactivities and more than half of them, including compounds such as aspeterreurone A [6], botryotins A–H [7] and penixylarins A–D [8], show cytotoxicity against different human cancer cell lines.

**Figure 1:**
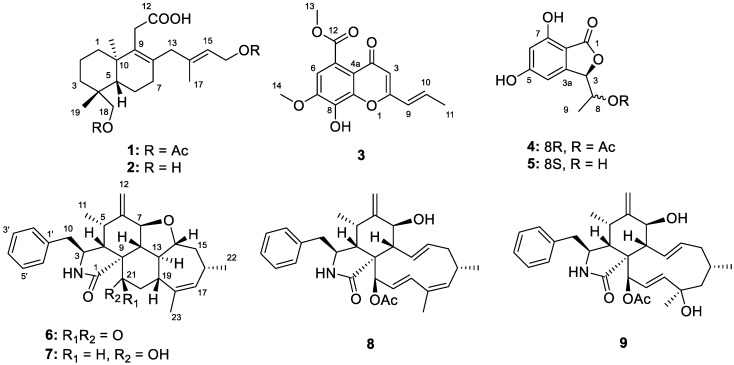
Chemical structures of **1**–**9**.

Our group has focused on the discovery of bioactive secondary metabolites from deep-sea-derived fungi for over ten years. In our previous study, a number of bioactive compounds with unprecedented skeletons were discovered in deep-sea-fungi from the South China Sea and Indian Ocean, such as the highly oxygenated tenellone-macrolide conjugated dimers lithocarpins A–D [9] and multi-cyclic meroterpenoids phomeroids A and B [10], both of which displayed significant cytotoxicity against human cancer cell lines. In this study, the chemical and biological investigation of a deep-sea-derived fungus *Diaporthe longicolla* FS429 was carried out and six new secondary metabolites (compounds **1**–**4**, **6** and **8**) together with three known compounds **5**, **7**, **9** were isolated (Figure 1). Compounds **4**, **6**–**9** exhibited antiproliferative effects against the four tested human tumor cell lines, while compounds **1** and **8** showed weak inhibitory activity against *Mycobacterium tuberculosis* protein tyrosine phosphatase B (*M*ptpB). Herein, the details of the isolation, structure identification and bioactivities of all these compounds are discussed.

## 2. Results and Discussion

### 2.1. Structure Elucidation of the New Compounds

The methanol extract of the fungus *Diaporthe longicolla* FS429 was concentrated under reduced pressure and further subjected to repeated column chromatography and semi-preparative HPLC to obtain the nine metabolites **1**–**9**.

Longidiacid A (**1**) was obtained as colorless powder. The molecular formula was deduced to be C_24_H_36_O_6_ based on the sodium adduct ion peak at *m*/*z* 443.2410 [M + Na]^+^ from HRESIMS (calcd for C_24_H_36_O_6_Na, 443.2404). The ^1^H-NMR data listed in Table 1 indicated the presence of five singlet methyls at *δ*_H_ 0.99 (6H, H_3_-19 and H_3_-20), 1.64 (3H, H_3_-17) and 2.06 (6H, OAc-16 and OAc-18); nine sp^3^ hybrid methylenes, including three characteristic AB coupling ones at *δ*_H_ 3.11/2.97 (d, *J* = 17.4, H_2_-11), 2.82/2.51 (d, *J* = 16.0, H_2_-13), 4.24/3.92 (d, *J* = 11.0, H_2_-18); a trisubstituted olefin proton at *δ*_H_ 5.24 (t, *J* = 6.9) and a sp^3^ methine (*δ*_H_ 1.40, H-5). The ^13^C-NMR spectrum resolved 24 carbons composed of three carbonyl carbons, four olefin carbons and 17 sp^3^ hybrid ones. According to a comprehensive analysis of the 1D NMR data and the degrees of unsaturation, compound **1** should be an acetylated bicyclic diterpenoid.

**Figure 2:**
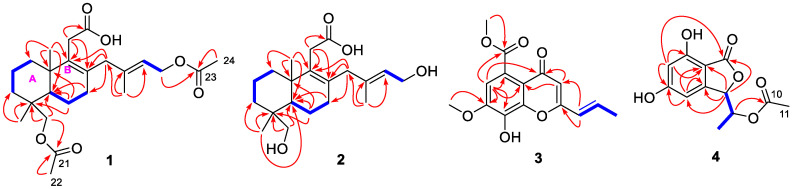
COSY (blue bold lines) and key HMBC correlations (red arrows) of **1**–**4**.

Analysis of the COSY spectrum (Figure 2) revealed the presence of three independent coupling fragments in **1** (C-1 to C-2 to C-3, C-5 to C-6 to C-7 and C-15 to C-16). The HMBC correlation from H_2_-18/H_3_-19 to C-3/C-4/C-5 and from H_3_-20 to C-1/C-5/C-10 constructed ring A in the structure. The ring B was elucidated based on the correlations from H_2_-11 to C-8/C-9/C-10 and from H_2_-6 to C-4/C-5/C-10. Moreover, the HMBC cross-peaks from H_3_-17 to C-13/C-14/C-15 as well as H_2_-13 to C-7/C-8/C-9 revealed an additional isopentenyl C_5_ unit (C-13 to C-17) connected to C-8. Finally, the carboxyl group at C-11 was evidenced by the correlation from H_2_-11 to C-12, while the two acetyl groups should be linked to their corresponding hydroxymethyls (C-16 and C-18).

**Figure 3:**
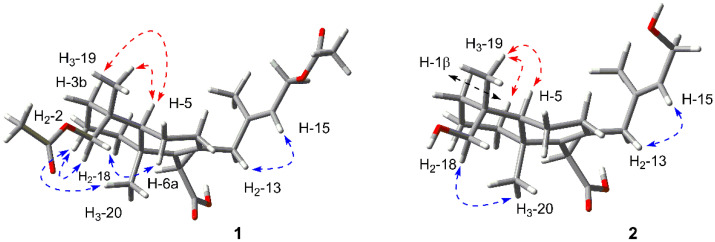
Key NOESY correlations of **1** and **2**.

The relative configuration was deduced by the NOESY analysis (Figure 3). The cross-peaks of H_2_-2/H_2_-18/H_3_-20 indicated that the Me-20 and methylene (H_2_-18) were *α*-oriented. H-5 and Me-19 were identified to be *β*-oriented by the correlations of H-18b/H-6a and H-5/H-3b/H_3_-19. The deficiency of the NOE effect between H-5 and H-18a or H-18b also supported the conclusion. Additionally, the correlation between H-15 and H_2_-13 suggested the *E* configuration of Δ^14^.

**Figure 4:**
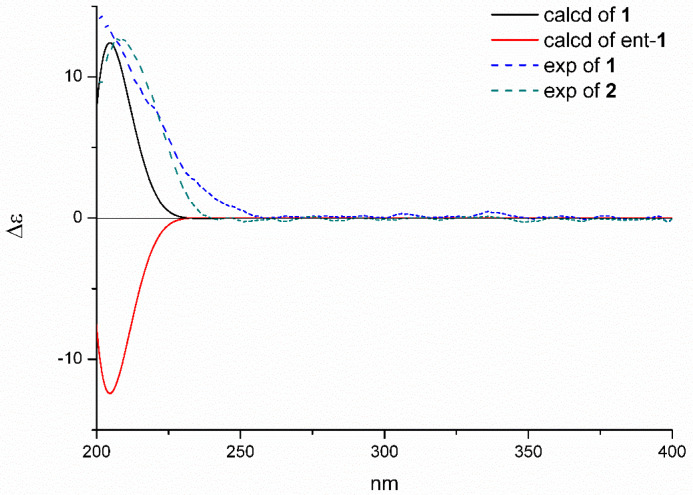
The calculated ECD spectra of **1**/ent-**1** at B3LYP/6-311+G(d,p) level and the experimental plot of **1/2**.

By comparing the experimental ECD spectrum of **1** with that calculated at the B3LYP/6-311+G(d,p) level (Figure 4, both of which exhibited a positive Cotton effect at 210 nm), the absolute configuration could be assigned as 4*R*, 5*S*, 10*R*.

Longidiacid B (**2**) was obtained as a colorless powder. The molecular formula was established to be C_20_H_32_O_4_ through the sodium adduct ion peak at *m*/*z* 359.2189 [M + Na]^+^ from HRESIMS (calcd for C_20_H_32_O_4_Na, 359.2193). The similar ^1^H- and ^13^C-NMR data listed in Table 1 indicated that it had a similar bicyclic diterpenoid core as longidiacid A. The main difference was the obvious shielded shift of H_2_-16 and H_2_-18, which suggested that compound **2** was a deacetylation product of **1**. Further analysis of the COSY and HMBC spectra (Figure 2) confirmed the planar structure of **2**.

The relative configuration was deduced by the NOESY analysis (Figure 3). The cross-peaks of H_2_-18/H_3_-20 suggested the *α*-orientation of them, while the correlations of H-5/H-1*β*/H_3_-19 indicated that the Me-19 and H-5 were *β*-oriented. Thus, the relative configuration was elucidated to be the same as that of **1**. Furthermore, the absolute configuration of **2** was finally assigned to be 4*R*, 5*S* and 10*R* based on the identical experimental ECD spectrum compared to that of **1**.

Longichromone A (**3**) was obtained as colorless powder, of which the molecular formula was assigned to be C_15_H_14_O_6_ based on the sodium adduct ion peak at *m*/*z* 313.0683 [M + Na]^+^ from HRESIMS (calcd for C_15_H_14_O_6_Na, 313.0683). The ^1^H-NMR data (Table 2) indicated the presence of a doublet doublet methyl at *δ*_H_ 1.98 (dd, *J* = 6.9 and 1.7, H_3_-11); four olefin methines including a *trans*-coupled double (*δ*_H_ 6.18 and 6.93) and two singlet ones (*δ*_H_ 6.06 and 6.94). The ^13^C-NMR spectrum resolved 15 carbons including of three methyls, a phenyl, two double bonds and two carbonyl carbons.

An acryl moiety (C-9 to C-11) was elucidated through the cross-peaks of H-9/H-10/H_3_-11 in COSY spectrum (Figure 2). By comparing the characteristic chemical shifts (*δ*_C_ 148.9, 134.9 and 144.3) with those of the known compound aspergchromone B [11], the benzene ring should be 1,2,3-*tri*-hydroxyl-substituted. The HMBC correlations from H-6 to C-4a/C-5/C-7/C-8, from H-3 to C-2/C-4/C-4a as well as the deshielded chemical shift of C-2 (*δ*_C_ 161.3) confirmed the chromone moiety, which was further supported by the weak four-bond correlation from H-6 to C-4. An *ortho*-substituted carboxyl group located at C-5 was deduced by the correlation from H-6 to C-12, meanwhile, the acryl was connected to C-2 based on the HMBC correlations from H-9 to C-2 and C-3. Finally, the correlations from H_3_-13 to C-12 and from H_3_-14 to C-7 revealed the substitution position of the two methoxy groups. By comprehensive comparison of the NMR data with those of aspergchromone B, compound **3** should be a dehydration product at C-10. Hence, the gross structure of **3** was established as shown.

Longiphthalidin A (**4**) was obtained as a colorless powder with the molecular formula of C_12_H_12_O_6_, which was deduced by the sodium adduct ion peak at *m*/*z* 275.0532 [M + Na]^+^ from HRESIMS (calcd for C_12_H_12_O_6_Na, 275.0526). The ^1^H-NMR data revealed the presence of two meta-coupling aromatic protons at *δ*_H_ 6.32 (d, *J* = 1.8, H-6) and 6.42 (m, H-4); two methines at *δ*_H_ 5.43 (d, *J* = 3.1, H-3) and *δ*_H_ 5.38 (dq, *J* = 3.1, 6.5, H-8); two methyls at *δ*_H_ 1.33 (d, *J* = 6.5, H_3_-9) and 6.94 (s, H_3_-11). The ^13^C-NMR spectrum resolved 12 carbons composing of a tetrasubstituted phenyl, two methines, two methyls and two ester carbonyl carbons (*δ*_C_ 170.2 and 170.1). Comprehensive analysis of 1D NMR data suggested that compound **4** was a derivative of acetophthalidin [12].

A meta-coupled benzene ring was deduced based on the HMBC correlations from H-4 to C-5/C-3a/C-7a, from H-6 to C-5/C-7/C-7a. The isobenzofuranone moiety was established by the correlations (Figure 2) from H-3 to C-1/C-7a/C-4/C-3a as well as the four-bonded correlation from H-6 to C-1. The coupling fragment from C-3 to C-9 was deduced through the cross-peaks of H-3/H-8/H_3_-9 in COSY spectrum. Moreover, an acetyl group was connected to C-8 based on the detected correlation between H-8 and C-10. Hence, the planar structure was completed.

The relative configuration of C-3 and C-8 was directly deduced by comparing the H-H coupling constant. Based on the previously published reference [12,13], the configuration of (3R*, 8S*)-**4** exhibited a *J*_3,8_ value of 4.5 Hz while the (3R*, 8R*)-**4** showed a lower value of 3.0 Hz. The *J*_3,8_ detected in compound **4** was 3.1 Hz, which suggested a 3R*, 8R* configuration. Finally, by comparing the same negative optical rotation value (${\left[\alpha \right]}_{D}^{25}$ −37) with that of the reported compound (*R*,*R*)-5,7-dihydroxy-3-(1-hydroxyethyl)-phthalide (${\left[\alpha \right]}_{D}^{25}$ −40) [12], we confirmed the absolute configuration was 3*R*, 8*R*.

**Figure 5:**
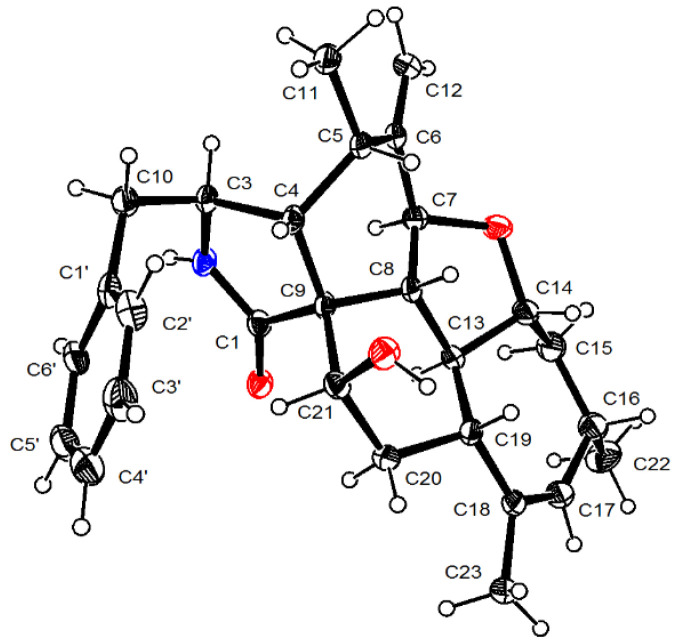
X-ray diffraction analysis of compound **7**.

Longichalasin A (**6**) was obtained as a colorless powder. The molecular formula was deduced to be C_28_H_33_O_3_N based on the protonated ion peak at *m*/*z* 432.2535 [M + H]^+^ from HRESIMS (calcd for C_28_H_34_NO_3_, 432.2533), indicating 13 degrees of unsaturation. The ^1^H-NMR data (Table 3) resolved the resonances of three methyls, four methylenes (including a terminal alkenyl), a single-substituted phenyl, 10 methines including an olefinic one. ^13^C-NMR data combined with HSQC spectra indicated the presence of two carbonyl groups and three quaternary carbons. All the above evidences implied that **6** might be a highly cyclized cytochalasin derivative. By comparing its NMR data with those of **7**, (cytochalasin J3 [14], previously reported from an Australian marine sediment-derived *Phomopsis* sp. and firstly confirmed through X-ray diffraction in this study, Figure 5), it could be concluded that the 21-OH in **7** was transferred to a keto-carbonyl in **6**.

**Figure 6:**
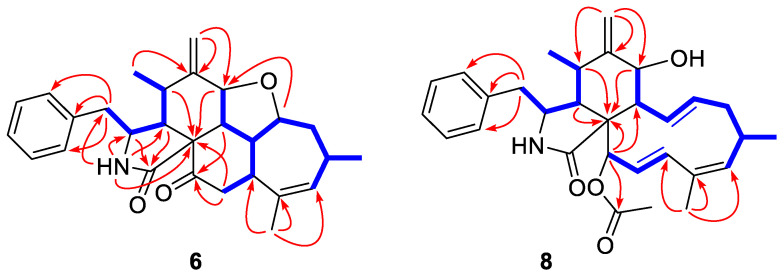
COSY (blue bold lines) and key HMBC correlations (red arrows) of **6** and **8**.

The COSY correlations of H_2_-10/H-3/H-4/H-5/H_3_-11 and H-7/H-8/H-13(/H-19/H_2_-20)/H-14/H_2_-15/H-16 (/H_3_-22)/H-17 revealed the two large coupling fragments. Meanwhile, the key HMBC correlations shown in Figure 6 constructed the pentacyclic skeleton. The correlations from H-8/H-9/H_2_-20 to C-21 confirmed the presence of the ketone carbonyl.

**Figure 7:**
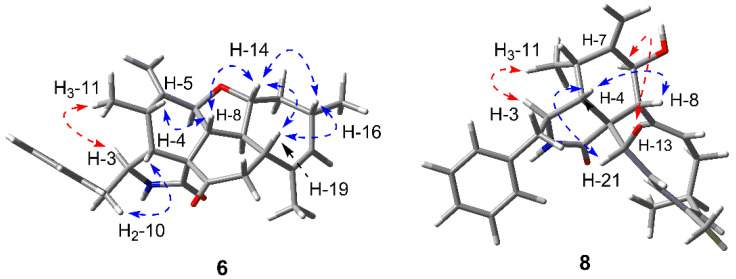
Key NOESY correlations of **6** and **8** (red and blue arrows implied the *α*- and *β*-orientation, respectively).

The relative configuration was deduced by the NOESY correlations (Figure 7) and H-H coupling constants. Firstly, the large coupling constant between H-13 and H-8/H-14/H-19 (12.4, 12.4, 9.8 Hz, respectively) indicated all of them laid in axal bond. The NOE correlations of H-5/H-8/H-14/H-16/H-19 demonstrated they were all *β*-oriented and the H-13 was *α*-oriented contrarily. Secondly, the large *J* value between H-7 and H-8 (12.4 Hz) suggested the *trans* configuration of them, and the relatively small *J* value between H-4/H-5 (4.1 Hz) evidenced the *β*-orientation of H-4. Finally, H-3 was assigned to be *α*-oriented based on the NOE correlations of H-3/H_3_-11 as well as H-4/H_2_-10.

**Figure 8:**
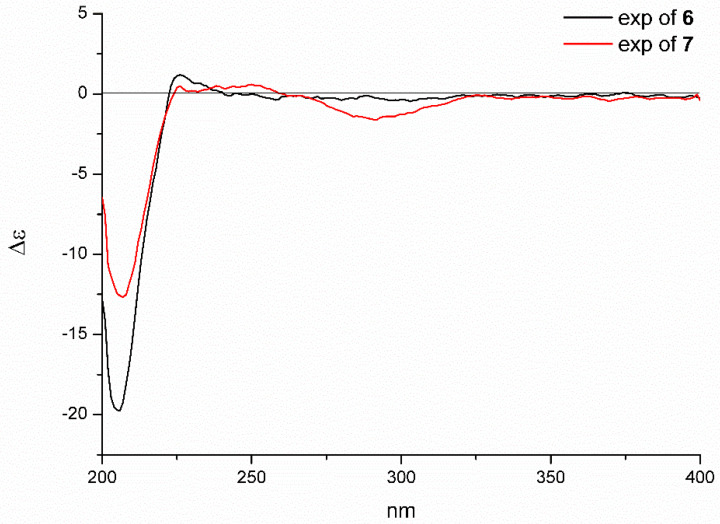
The experimental ECD spectra of **6** and **7**.

Since compounds **6** and **7** exhibited the nearly identical ECD spectrum (Figure 8), the absolute configuration of **6** could be assigned as 3*S*, 4*S*, 5*S*, 7*S*, 8*S*, 9*R*, 13*S*, 14*R*, 16*S*, 19*R*.

Longichalasin B (**8**) was obtained as a colorless powder, of which the molecular formula was deduced to be C_30_H_37_O_4_N by the protonated ion peak at *m*/*z* 476.2802 [M + H]^‒^ from HRESIMS (calcd for C_30_H_38_NO_4_, 476.2795). By comparing the 1D NMR data with those reported previously, it could be concluded that compound **8** was closely related to the metabolite cytochalasin H (**9**) [15] except for the absence of a -C(OH)-CH_2_- fragment and the presence of an additional double bond (Δ^17^), which suggested that the hydroxyl group at C-17 was dehydrated. The COSY cross-peaks (Figure 6) of H-17/H-18 as well as the HMBC correlations from H_3_-23 to C-17/C-18/C-19 indicated the location of Δ^17^. The acetyl group was linked to the hydroxyl group at C-21, which was deduced by the correlation from H-21 to the carboxyl carbon of acetyl group. Thus, the planar structure was established as shown.

The relative configuration was deduced by analysis of the NOESY spectrum (Figure 7) as well as the H-H coupling constants. The NOE correlation between H-4 and H-8 indicated the *β*-orientation of them. The large *J* value (10.6 Hz) suggested the *trans*-diaxial designation of H-7 and H-8, which laid H-7 to *α*-orientation. The key correlation between H-7 and H-13 also confirmed the conclusion. Furthermore, the NOE cross-peak between H-4 and H-21 evidenced the *α*-oriented of the amide ring, while the correlation between H-3 and H_3_-11 indicated the coplanarity of them. Finally, by comparing the same positive optical rotation values of compound **9** (${\left[\alpha \right]}_{D}^{25}$ +29) as well as the similar ECD data (both exhibited negative Cotton effects at 215 nm and 300 nm), the absolute configuration of **8** was assigned to be 3*S*, 4*S*, 5*S*, 7*S*, 8*S*, 9*R*, 16*S*, 19*R*.

### 2.2. Bioactivities

Compounds **1**–**9** were evaluated for their in vitro cytotoxicity against four human cancer cell lines (SF-268, MCF-7, HepG-2 and A549). Longichalasin B (**8**) exhibited antiproliferative effect against SF-268 with the IC_50_ value of 16.44 μM. (Table 4). Besides, compounds **4**, **6**, **7** and **9** also displayed marginal activities at the concentration of 100 μM.

Moreover, the *Mycobacterium tuberculosis* protein tyrosine phosphatase B (*M*ptpB) inhibitory activity of **1**–**9** was also tested. However, only compounds **1** and **8** were detected to show weak inhibitory effects (inhibited 35.4% and 53.5% of enzyme activity at a concentration of 50 μM).

## 3. Materials and Methods

### 3.1. General Experimental Procedures

Optical rotations were measured using an Anton Paar MCP-500 instrument (Anton Paar, Graz, Austria) and the circular dichroism (ECD) as well as the UV spectra were collected on a Jasco 820 spectropolarimeter (JASCO, Tokyo, Japan) in the 200–400 nm range (under N_2_ protection). Infrared (IR) spectra were recorded on an IR Affinity-1 spectrophotometer (Shimadzu, Kyoto, Japan). All the 1D and 2D NMR data were recorded on a Avance-III 600 MHz HD spectrometer (Bruker, Bremen, Germany) with tetramethylsilane as an internal standard. HR-ESI-MS were collected on Bruker maXis high resolution mass spectrometer. A Shimadzu LC-20 AT equipped with an SPD-M20A PDA detector was used for HPLC analysis and preparative separations. An ACE 5 PFP-C_18_ column (250 × 10.0 mm, 5 μm, 12 nm) was used for semipreparative HPLC separation, meanwhile, a CHIRAL-MD (2)-RH column (250 × 10.0 mm, 5 μm) was used for chiral-phase chromatography (Guangzhou FLM Scientific Instrument Co., Ltd., Guangzhou, China). Column chromatography material: commercial silica gel (200–300 mesh) was purchased from Qingdao Marine Chemical Plant (Qingdao, China); Sephadex LH-20 gel was purchased from Amersham Biosciences, Shanghai, China). All analytical grade solvents were purchased from Guangzhou Chemical Regents Company (Guangzhou, China). The natural sea salt was produced by Guangdong Yueyan saltern (Guangdong, China).

### 3.2. Fungal Material

The strain FS429 investigated in this research was identified to be *Diaporthe longicolla*, which was collected from the deep-sea sediment in the Indian Ocean (4°0.188′ N, 90°44.909′ E; depth 3000 m) in March 2016. The fungal identification was proceeded according to morphological traits and ITS rDNA sequence analysis. The sequence data have been submitted to GenBank, under accession number MT678558. The strain FS429 was now deposited at the Guangdong Provincial Key Laboratory of Microbial Culture Collection and Application, Guangdong Institute of Microbiology. Working stocks were prepared on PDA (potato 200 g/L, glucose 20 g/L, KH_2_PO_4_ 3 g/L, MgSO_4_•7H_2_O 1.5 g/L, vitamin B_1_ 10 mg/L, natural sea salt 15 g/L) slants stored at 4 °C.

### 3.3. Fermentation, Extraction, and Isolation

A grown plate culture of *Diaporthe longicolla* FS429 was prepared for the seed cultures. After the mycelia being inoculated in PDB culture at 28 °C for 5 days in a rotary shaker (200 rpm), it was transferred into the rice solid medium (15 Erlenmeyer flasks each containing 250 g rice and 400 mL H_2_O with 3% natural sea salt) and incubated at room temperature for another 28 days. Then, the solid fermented substrate was extracted with methanol for three times to yield a dark brown oily residue (105.4 g). After subjected to silica gel column chromatography eluting with petroleum ether/EtOAc in a linear gradient (10:1 to 1:1), 36 fractions (Fr.1–Fr.36) were obtained. Fr.23 was subjected to Sephadex LH-20 eluting with MeOH/CH_2_Cl_2_ (1:1) to obtain five fractions (Fr.23.1–Fr.23.5) and the Fr.23.3 was further purified by HPLC with PFP-C_18_ column (MeOH/H_2_O, 80:20, 2 mL/min) to give **1** (7.3 mg, *t*_R_ = 9.9 min) and **2** (2.1 mg, *t*_R_ = 11.0 min). The Fr.13 was subjected to repeated silica gel column and purified by Sephadex LH-20 to yield **3** (5.9 mg), **4** (3.3 mg) and **5** (12.1 mg). The fraction Fr.17 was separated directly by HPLC equipped PFP-C_18_ column (MeOH/H_2_O, 80:20, 2 mL/min) to obtain two pairs of mixtures. After purified by chiral-phase HPLC with a CHIRAL-MD (2)-RH column (flow rate = 2 mL/min; mobile phase 70% MeCN/H_2_O, the pure **6** (3.1 mg, *t*_R_ = 13.9 min), **7** (14.0 mg, *t*_R_ = 14.8 min), **8** (8.2 mg, *t*_R_ = 15.6 min) and **9** (17.4 mg, *t*_R_ = 16.9 min) were obtained.

#### 3.3.1. Longidiacid A (**1**)

Colorless powder; ${\left[\alpha \right]}_{D}^{25}$ +3.8 (*c* 0.10, MeOH); CD (0.30 mg/mL, MeOH): 210 (+4.44) nm; UV (MeOH) *λ*_max_ (log *ε*): 202 (4.00), 247 (3.34) nm; IR (KBr): 3320, 2929, 1727, 1701, 1450, 1260, 1112 cm^−1^; ^1^H- and ^13^C-NMR data, Table 1 and Figures S1 and S2. HRESIMS *m/z* 443.2410 [M + Na]^+^ (calcd for C_24_H_36_O_6_Na, 443.2404).

#### 3.3.2. Longidiacid B (**2**)

Colorless powder; ${\left[\alpha \right]}_{D}^{25}$ +2.0 (*c* 0.10, MeOH); CD (0.30 mg/mL, MeOH): 211 (+4.43) nm; UV (MeOH) *λ*_max_ (log *ε*): 203 (3.77) nm; IR (KBr): 3324, 2933, 1704, 1451, 1255, 1109 cm^−1^; ^1^H- and ^13^C-NMR data, Table 1 and Figures S7 and S8. HRESIMS *m/z* 359.2189 [M + Na]^+^ (calcd for C_20_H_32_O_4_Na, 359.2193).

#### 3.3.3. Longichromone A (**3**)

Colorless powder; UV (MeOH) *λ*_max_ (log *ε*): 210 (4.01), 259 (4.08), 328(3.86) nm; IR (KBr): 3334, 1720, 1651, 1396, 1109, 980, 855 cm^−1^; ^1^H- and ^13^C-NMR data, Table 2 and Figures S13 and S14; HRESIMS *m*/*z* 313.0683 [M + Na]^+^ (calcd for C_15_H_14_O_6_Na, 313.0683).

#### 3.3.4. Longiphthalidin A (**4**)

Colorless powder; ${\left[\alpha \right]}_{D}^{25}$ ‒37 (*c* 0.10, MeOH); UV (MeOH) *λ*_max_ (log *ε*): 219 (4.23), 263 (3.94) nm; IR (KBr): 3454, 1725, 1620, 1470, 1200, 1026, 997 cm^−1^; ^1^H- and ^13^C-NMR data, Table 2 and Figures S18 and S19; HRESIMS *m*/*z* 275.0532, [M + Na]^+^ (calcd for C_12_H_12_O_6_Na, 275.0526).

#### 3.3.5. Longichalasin A (**6**)

Colorless powder; ${\left[\alpha \right]}_{D}^{25}$ +9 (*c* 0.10, MeOH); CD (0.33 mg/mL, MeOH): 206 (−4.12), 290 (−2.67) nm; UV (MeOH) *λ*_max_ (log *ε*): 209 (4.08), 260 (2.82) nm; IR (KBr): 3430, 2959, 2920, 1719, 1688, 1452, 1418, 1307, 1060, 1030 cm^−1^; ^1^H- and ^13^C-NMR data, Table 3 and Figures S23 and S24; HRESIMS *m*/*z* 432.2535 [M + H]^+^ (calcd for C_28_H_34_NO_3_, 432.2533).

#### 3.3.6. Longichalasin B (**8**)

Colorless powder; ${\left[\alpha \right]}_{D}^{25}$ +19 (*c* 0.1, MeOH); CD (0.33 mg/mL, MeOH): 216 (−4.31), 299 (−2.55) nm; UV (MeOH) *λ*_max_ (log *ε*): 208 (4.20) nm; IR (KBr): 3422, 3331, 2961, 2917, 1722, 1709, 1444, 1400, 1059, 998 cm^−1^; ^1^H- and ^13^C-NMR data, Table 3 and Figures S29 and S30. HRESIMS *m*/*z* 476.2802 [M + H]^+^ (calcd for C_30_H_38_NO_4_, 476.2795).

### 3.4. Details of Quantum Chemical Calculations

The Spartan’14 software (Wavefunction Inc., V1.1.0., Irvine, CA, USA) and the Gaussian 09 program were used to proceed the Merck molecular force field (MMFF) and DFT/TD-DFT calculations, respectively [16]. Conformers with an energy lower the 10 kcal mol^−1^ were generated and re-optimized using at the B3LYP/6-31+G(d,p) level, meanwhile, the frequency calculations were performed at the same level to confirm that each optimized conformer was a true minimum and to estimate their relative thermal free energy (ΔG) at 298.15 K. Finally, conformers displaying the Boltzmann distribution over 5% (Table S1) were subjected to ECD calculations at B3LYP/6-311+G(d,p) level (rotatory strengths were generated for a total of 20 excited states). Self-consistent reaction field (SCRF) method with the polarizable continuum model (PCM) was applied for solvent effects. The ECD spectrum was generated by the SpecDis program [17] using a Gaussian band shape with 0.30 eV exponential half-width from dipole-length dipolar and rotational strengths. 

### 3.5. Cytotoxicity Assay

The in vitro cytotoxicity assays were carried out according to our previously reported method [18]. SF-268, MCF-7, HepG-2 and A549 were selected to be the targeted cancer cell lines. All the cells were cultivated in RPMI 1640 medium and detached with 0.1% trypsin-EDTA. The four tested cell lines were injected into 96-well plates and incubated at 37 °C for 24 h under 5% CO_2_ protection. Then, different concentrations of the inhibitors were added and further co-incubated for 72 h. After stained, cell monolayers were fixed with 50 μL trichloroacetic acid (*wt/v*: 50%) and stained with 0.4% SRB (dissolved in 1% CH_3_COOH) for 30 min. The monolayers were washed by 1% CH_3_COOH three times to remove the unbound dye. Cell monolayers were fixed with 50 μL trichloroacetic acid (*wt/v*: 50%) and stained with 0.4% SRB (dissolved in 1% CH_3_COOH) for 30 min. The monolayers were washed by 1% CH_3_COOH three times to remove the unbound dye. Cisplatin was used as a positive control possessing potent cytotoxic activity. All data were obtained in triplicate. The human cancer cell lines SF-268, MCF-7, HepG-2, A549 were purchased from the Cell Bank of the Chinese Academy of Sciences (Shanghai, China).

### 3.6. MptpB Inhibitory Activity

The enzyme was prepared according to the method reported in the literature [19], and the details of the experiment was performed based on our previously reported literature [20]. The protein was purified from *E. coli* BL21 (DH3) with heterologously expressing the ptpB gene of the *M. tuberculosis* H37Rv strain (School of Life Sciences, Sun Yat-sen University, Guangzhou, China). The inhibitory assays were carried out using the RediPlate 96 EnzChek tyrosine phosphatase assay kit (Invitrogen, Waltham, MA, USA) by measuring the absorbance of the fluorogenic phosphatase substrate (6,8-difluoromethylumbelliferyl phosphate). Tested compounds with gradient concentrations and enzyme were dissolved in buffer and added into appropriate *M*ptpB buffer. After incubating at room temperature for 30 min, the plate was monitored at 355 and 460 nm immediately for 15 min. All measurements were carried out in triplicate. Oleanolic acid was used as positive control with an IC_50_ value of 14 μM.

## 4. Conclusions

In conclusion, six new secondary metabolites **1**–**4**, **6** and **8** together with three known compounds **5**, **7**, **9** were isolated from the methanol extract of the deep-sea derived fungus *Diaporthe longicolla* FS429. Their structures were established through comprehensive spectroscopic analysis, including ECD calculations, while the structure of compound **7** was confirmed through X-ray diffraction for the first time. In the bioassays, compounds **8** exhibited antiproliferative effect against SF-268 with the IC_50_ values of 16.44 μM. Besides, compounds **1** and **8** showed weak inhibitory activity against *Mycobacterium tuberculosis* protein tyrosine phosphatase B (*M*ptpB). This study will make contributions to the chemical and biological diversities of secondary metabolites from deep-sea-derived fungi.

## Figures and Tables

**Table 1:** The ^1^H and ^13^C NMR data of **1** and **2**.

Position	1 ^a^		2 ^a^	
	*δ*_C_, Type.	*δ*_H_ (*J* in Hz)	*δ*_C_, Type.	*δ*_H_ (*J* in Hz)
1	35.8, CH_2_	1.71, m	35.4, CH_2_	1.83, m
		1.74, m		1.80, m
2	18.4, CH_2_	1.49, m	18.2, CH_2_	1.58, m
				1.41, m
3	35.8, CH_2_	1.28, m	34.9, CH_2_	1.33, m
		1.05, dd (13.5, 4.8)		0.93, m
4	37.0, C		38.4, C	
5	51.9, CH	1.40, m	51.5, CH	1.50, m
6	19.0, CH_2_	1.82, dd (12.2, 5.9)	18.8, CH_2_	1.76, m
		1.46, m		1.44, m
7	31.5, CH_2_	2.02, m	31.1, CH_2_	2.02, m
8	131.8, C		128.7, C	
9	135.8, C		138.8, C	
10	38.7, C		38.6, C	
11	32.8, CH_2_	3.11, d (17.4)	36.2, CH_2_	2.96, d (16.9)
		2.97, d (17.4)		2.80, d (16.9)
12	178.3, C		179.9, C	
13	42.9, CH_2_	2.82, d (16.0)	42.4, CH_2_	2.94, d (16.1)
		2.51, d (16.0)		2.35, d (16.1)
14	139.6, C		136.5, C	
15	118.6, CH	5.24, t (6.9)	122.7, CH	5.28, t (6.8)
16	61.3, CH_2_	4.58, d (6.9)	58.1, CH_2_	4.07, d (6.8)
17	16.7, CH_3_	1.64, s	15.4, CH_3_	1.60, s
18	67.1, CH_2_	4.24, d (11.0)	63.7, CH_2_	3.77, d (11.9)
		3.92, d (11.0)		3.31, (overlap)
19	27.1, CH_3_	0.99, s	26.0, CH_3_	0.96, s
20	20.5, CH_3_	0.99, s	20.0, CH_3_	0.98, s
21	171.5, C			
22	21.1, CH_3_	2.06, s		
23	171.3, C			
24	21.1, CH_3_	2.06, s		

^a^ Recorded at 400 MHz (^1^H) and 100 MHz (^13^C) in CDCl_3_-*d*.

**Table 2:** The ^1^H and ^13^C NMR Data of **3** and **4**.

Position	3 ^a^		Position	4 ^b^	
	*δ*_C_, Type.	*δ*_H_ (*J* in Hz)		*δ*_C_, Type.	*δ*_H_ (*J* in Hz)
1	37.0, C		1	170.2, C	
2	161.3, C		3	81.0, CH	5.43, brd (3.1)
3	108.4, CH	6.06, s	3a	150.6, C	
4	176.9, C		4	100.9, CH	6.42, m
4a	116.1, C		5	165.4, C	
5	124.0, C		6	102.5, CH	6.32, d (1.8)
6	107.4, CH	6.94, s	7	158.2, C	
7	148.9, C		7a	103.8, C	
8	134.9, C		8	69.2, CH	5.38, dq (3.1, 6.5)
8a	144.3, C		9	14.6, CH_3_	1.33, d (6.5)
9	123.8, CH	6.18, dq (15.5, 1.7)	10	170.1, C	
10	137.2, CH	6.93, dq (15.5, 6.9)	11	19.3, CH_3_	1.89, s
11	18.7, CH_3_	1.98, dd (6.9, 1.7)			
12	170.0, C				
13	53.1, CH_3_	3.97, s			
14	56.8, CH_3_	4.01, s			-

^a^ Recorded at 400 MHz (^1^H) and 100 MHz (^13^C) in CDCl_3_-*d*; ^b^ Recorded at 400 MHz (^1^H) and 100 MHz (^13^C) in methanol-*d*_4_.

**Table 3:** The ^1^H and ^13^C NMR data of **6** and **8**.

Position	6 ^a^		8 ^a^	
	*δ*_C_, Type.	*δ*_H_ (*J* in Hz)	*δ*_C_, Type.	*δ*_H_ (*J* in Hz)
1	170.9, C		174.1, C	
2	-	5.45, brs	-	-
3	53.9, CH	3.34, dt (9.8, 4.1)	53.6, CH	3.25, m
4	43.3, CH	3.07, t (4.1)	50.6, CH	2.13, d (5.3, 3.2)
5	35.1, CH	2.87, m	32.6, CH	2.80, m
6	147.4, C		147.2, C	
7	76.3, CH	4.26, dd (12.4, 2.6)	69.6, CH	3.72, d (10.6)
8	52.1, CH	2.22, t (12.4)	47.7, CH	3.04, brt (10.1)
9	59.7, C		48.2, C	
10	45.2, CH_2_	2.95, dd (13.6, 4.1)	45.5, CH_2_	2.84, dd (13.5, 4.9)
		2.58, dd (13.6, 9.8)		2.70, dd (13.5, 9.6)
11	14.6, CH_3_	1.18, d (6.7)	13.5, CH_3_	0.98, d (6.7)
12	114.5, CH_2_	5.39, brt (2.4)	114.6, CH_2_	5.29, brs
		5.23, brt (2.5)		5.10, brs
13	44.4, CH	2.43, dt (12.4, 9.8)	130.4, CH	6.02, dd (15.6, 9.6)
14	87.3, CH	3.70, ddd (12.4, 11.8, 3.1)	137.2, CH	5.72, ddd (15.6, 10.8, 4.8)
15	39.8, CH_2_	2.01, dt (11.8, 3.1)	42.9, CH_2_	2.24, m
		1.47, q (11.8)		2.00, m
16	30.3, CH	2.13, m	31.7, CH	2.70, m
17	133.3, CH	5.27, brs	136.0, CH	5.26, brd (7.6)
18	137.3, C		132.5, C	
19	41.7, CH	2.60, m	136.1, CH	6.67, d (16.4)
20	43.3, CH_2_	3.47, dd (14.0, 12.4)	120.4, CH	5.61, dd (16.4, 3.7)
		2.73, dd (14.0, 5.1)		
21	204.9, C		78.1, CH	5.48, m
22	24.5, CH_3_	1.13, d (7.2)	24.0, CH_3_	1.05, d (6.9)
23	23.4, CH_3_	1.76, s	21.1, CH_3_	1.82, s
1′	137.4, C		137.4, C	
2′/6′	129.0, CH	7.15, brd (6.80)	129.1, CH	7.15, m
3′/5′	129.0, CH	7.32, m	128.9, CH	7.32, m
4′	127.1, CH	7.25, m	127.1, CH	7.26, m

^a^ Recorded at 400 MHz (^1^H) and 100 MHz (^13^C) in CDCl_3_-d.

**Table 4:** Cytotoxic Activity of the **1**–**9** against Different Human Cancer Cell Lines.

Compounds	IC_50_ (µM) ^a^			
	SF-268	MCF-7	HepG-2	A549
**1**	>150	>150	>150	>150
**2**	>150	>150	>150	>150
**3**	>150	>150	>150	>150
**4**	33.83 ± 2.43	88.95 ± 3.35	91.86 ± 8.74	88.25 ± 5.87
**5**	>150	>150	>150	>150
**6**	65.33 ± 1.59	73.48 ± 0.42	63.84 ± 2.73	64.00 ± 0.50
**7**	74.38 ± 6.24	79.55 ± 2.82	63.67 ± 1.25	70.29 ± 2.55
**8**	16.44 ± 0.75	36.45 ± 1.97	59.09 ± 1.30	33.34 ± 1.24
**9**	68.94 ± 2.15	91.91 ± 4.86	94.04 ± 2.56	84.52 ± 4.57
Cisplatin ^b^	3.18 ± 0.04	2.78 ± 0.15	2.21 ± 0.02	1.49 ± 0.02

^a^ Results are expressed as the mean ± standard error; ^b^ Positive control.

## Supplementary Materials

The following are available online at https://www.mdpi.com/1660-3397/18/8/381/s1, Figures S1–S34: The 1D and 2D NMR data of compounds **1**–**4**, **6** and **8**, Figures S35–S40: HRESIMS of compounds **1**–**4**, **6** and **8**, Table S1: B3LYP/6-31+G(d,p) optimized low-energy conformers of **1**.
