# Supplementary material for: Bioactive Metabolites from the Deep-Sea-Derived Fungus Diaporthe longicolla FS429
Source: Mar Drugs. 2020 Jul 23;18(8):381. doi: 10.3390/md18080381 (PMC7460381; doi:10.3390/md18080381)
Supplement: Supplementary file 1 [file marinedrugs-18-00381-s001.pdf]

SUPPORTING INFORMATION

[illegible]active Metabolites from the Deep-Sea-Derived Fungus *Diaporthe longicolla* FS429: Isolation, Structure Identification and Bioactivities

Zhaoming Liu,[†] Yuchan Chen,[†] Saini Li,[†] Qinglin Wang,[‡] Caiyun Hu,[†] Hongxin Liu,[*,†] Weimin, Zhang[*,†]

[†]*State Key Laboratory of Applied Microbiology Southern China, Guangdong Provincial Key Laboratory of Microbial Culture Collection and Application, Guangdong Open Laboratory of Applied Microbiology, Guangdong Institute of Microbiology, Guangdong Academy of Sciences, 100 Central Xianlie Road, Yuexiu District, Guangzhou 510070, China*;

[‡]*School of Life Sciences and Biomedical Center, Sun Yat-Sen University, Guangzhou 510275, PR China*;

Table of contents:

Figure S1. ^{1}H NMR spectrum of **1** in $CDCl_3$.
Figure S2. ^{13}C NMR spectrum of **1** in $CDCl_3$.
Figure S3. ^{1}H, ^{1}H-COSY spectrum of **1** in $CDCl_3$.
Figure S4. HSQC spectrum of **1** in $CDCl_3$.
Figure S5. HMBC spectrum of **1** in $CDCl_3$.
Figure S6. NOESY spectrum of **1** in $CDCl_3$.
Figure S7. ^{1}H NMR spectrum of **2** in CD_3OD.
Figure S8. ^{13}C NMR spectrum of **2** in CD_3OD.
Figure S9. ^{1}H, ^{1}H-COSY spectrum of **2** in CD_3OD.
Figure S10. HSQC spectrum of **2** in [illegible]OD.
Figure S11. HMBC spectrum of **2** in CD_3OD.
Figure S12. NOESY spectrum of **2** in CD_3OD.
Figure S13. ^{1}H NMR spectrum of **3** in $CDCl_3$.
Figure S14. ^{13}C NMR spectrum of **3** in $CDCl_3$.
Figure S15. ^{1}H, ^{1}H-COSY spectrum of **3** in $CDCl_3$.
Figure S16. HSQC spectrum of **3** in $CDCl_3$.
Figure S17. HMBC spectrum of **3** in $CDCl_3$.
Figure S18. ^{1}H NMR spectrum of **4** in CD_3OD.
Figure S19. ^{13}C NMR spectrum of **4** in CD_3OD.
Figure S20. ^{1}H, ^{1}H-COSY spectrum of **4** in CD_3OD.
Figure S21. HSQC spectrum of **4** in CD_3OD.
Figure S22. HMBC spectrum of **4** in CD_3OD.
Figure S23. ^{1}H NMR spectrum of **6** in $CDCl_3$.
Figure S24. ^{13}C NMR spectrum of **6** in $CDCl_3$.
Figure S25. ^{1}H, ^{1}H-COSY spectrum of **6** in $CDCl_3$.
Figure S26. HSQC spectrum of **6** in $CDCl_3$.
Figure S27. HMBC spectrum of **6** in $CDCl_3$.
Figure S28. NOESY spectrum of **6** in $CDCl_3$.
Figure S29. ^{1}H NMR spectrum of **8** in $CDCl_3$.
Figure S30. ^{13}C NMR spectrum of **8** in $CDCl_3$.
Figure S31. ^{1}H, ^{1}H-COSY spectrum of **8** in $CDCl_3$.
Figure S32. HSQC spectrum of **8** in $CDCl_3$.
Figure S33. HMBC spectrum of **8** in $CDCl_3$.
Figure S34. NOESY spectrum of **8** in $CDCl_3$.
Figure S35. HR-ESI-MS spectrum of **1**.
Figure S36. HR-ESI-MS spectrum of **2**.
Figure S37. HR-ESI-MS spectrum of **3**.
Figure S38. HR-ESI-MS spectrum of **4**.
Figure S39. HR-ESI-MS spectrum of **6**.
Figure S40. HR-ESI-MS spectrum of **8**.
Table S1. Energy analysis for the Conformers of **1**.

Data were collected on an Agilent Xcalibur Nova single-crystal diffractometer using Cu Kα radiation. The crystal structure was refined by full-matrix least-squares calculation with the SHELXL-97. Crystallographic data for the structure of 1 have been deposited in the Cambridge Crystallographic Data Centre (deposition number: CCDC 2013296). Crystal data of 1: $C_{28.5}H_{36}O_3NCl$ (M = 476.03); needle crystal (0.2 × 0.1 × 0.1); space group P21; unit cell dimensions a = 8.6225(1) Å, b = 46.1799(8) Å, c = 13.4858(2) Å, α = 90°, β = 108.263(2)°, γ = 90°, V = 5099.37(14) Å3, Z = 8; T = 150(2) K; ρcald = 1.240 mg/m^3; absorption coefficient 1.555 mm−1; F(000) = 2040, a total of 84002 reflections were collected in the range 6.90° < θ < 74.76°, independent reflections 19729 [R(int) = 0.0731]; the number of data/parameters/restraints were 6315/6/475; goodness-offit on F2 = 1.025; final R indices [I > 2σ(I)] R_1 = 0.0723, ωR_2 = 0.1932; R indices (all data) R_1 = 0.0757, ωR_2 = 0.1965. Flack parameter 0.070 (11).

Figure S1. ^{1}H NMR spectrum of **1** in $CDCl_3$.

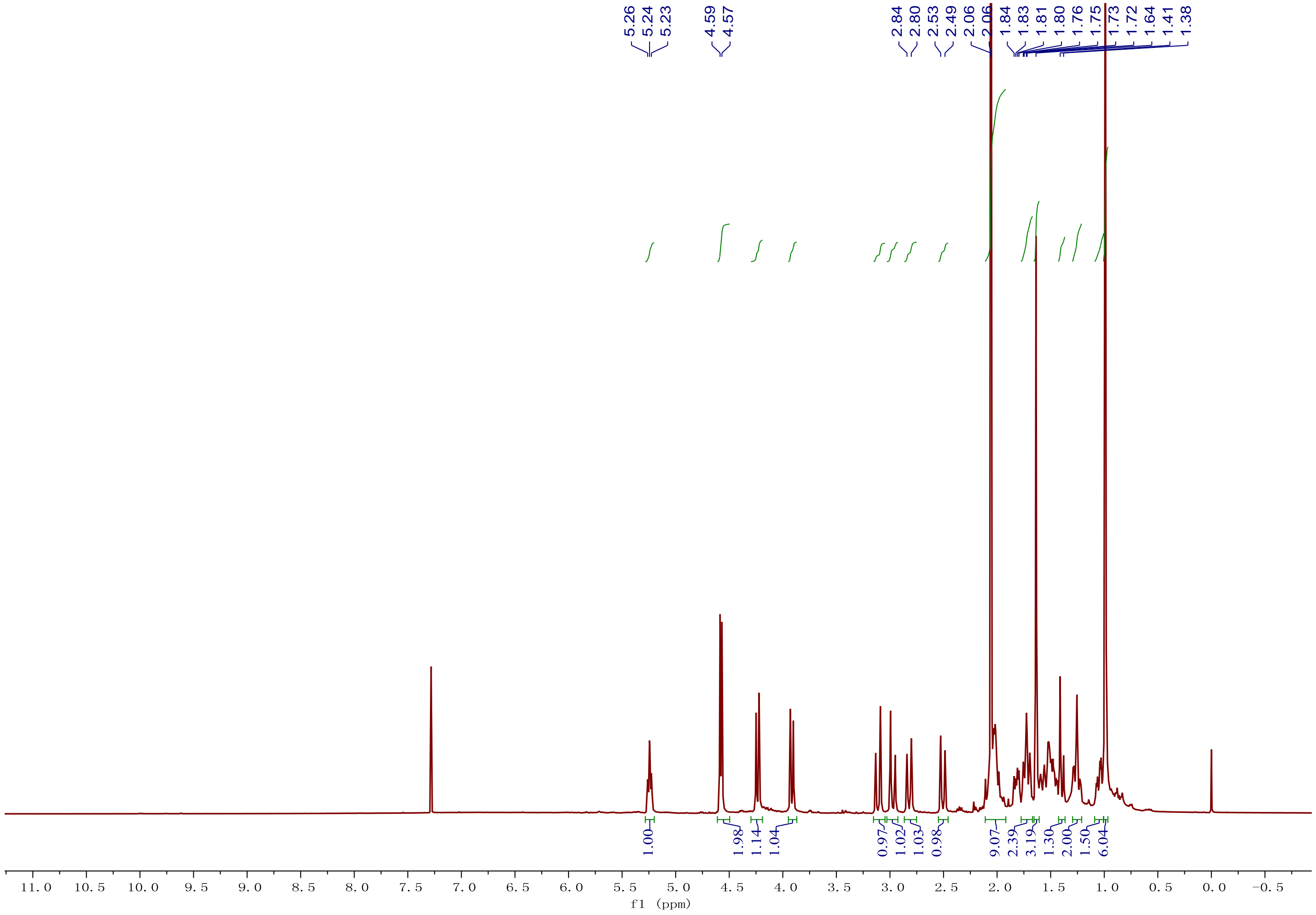

Figure S2. ^{13}C NMR spectrum of **1** in $CDCl_3$.

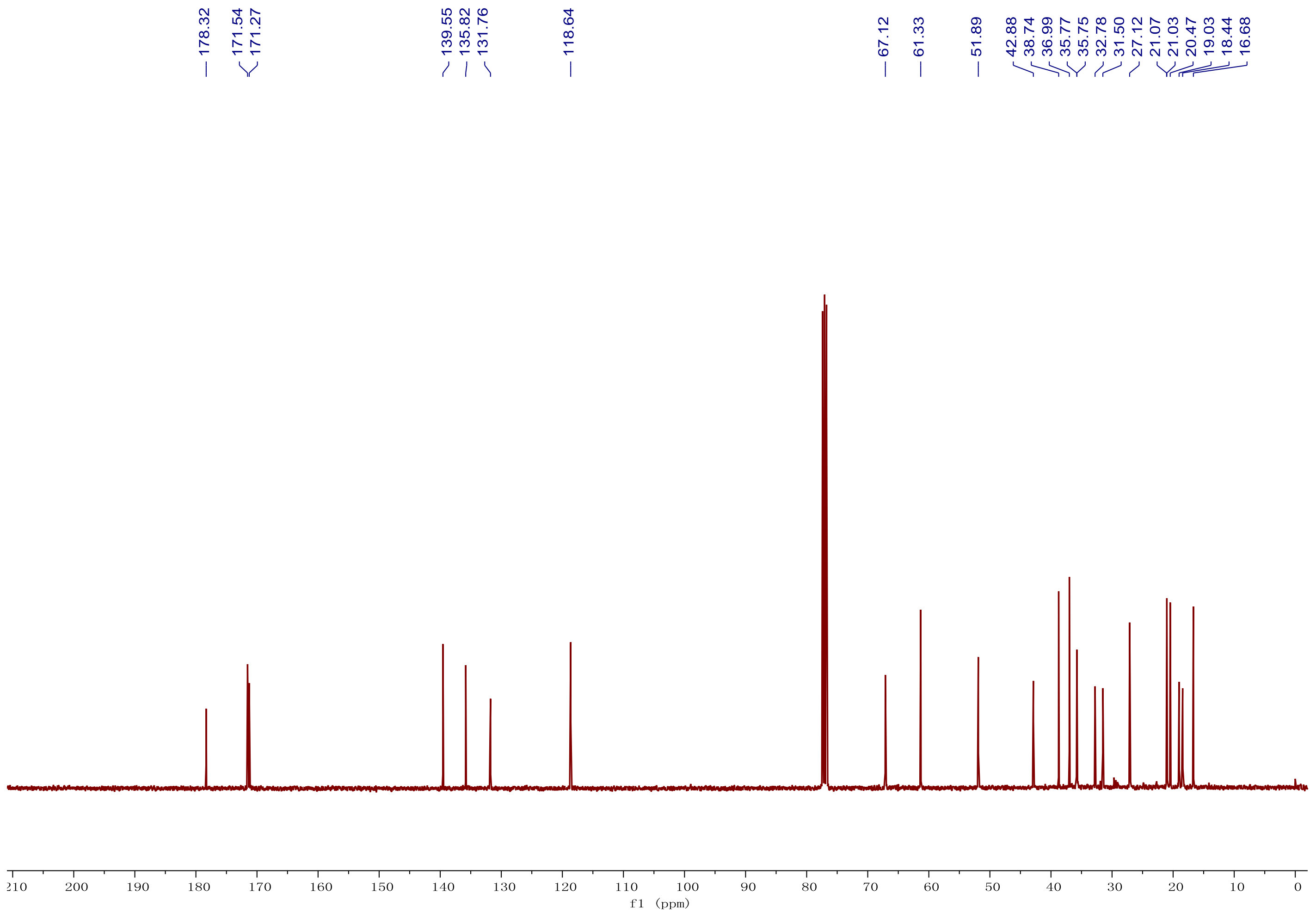

Figure S3. ^{1}H, ^{1}H-COSY spectrum of **1** in $CDCl_3$.

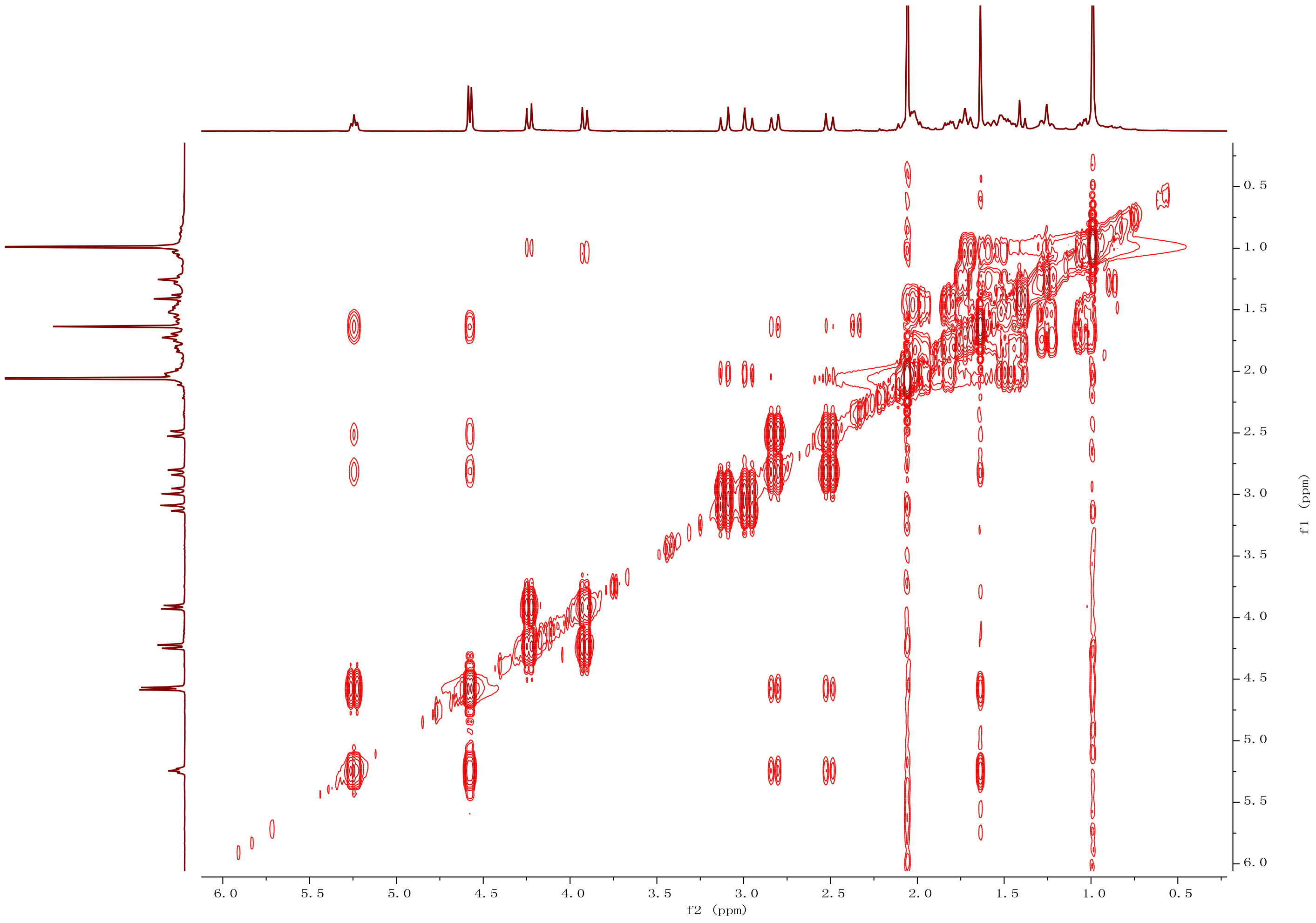

Figure S4. HSQC spectrum of **1** in $CDCl_3$.

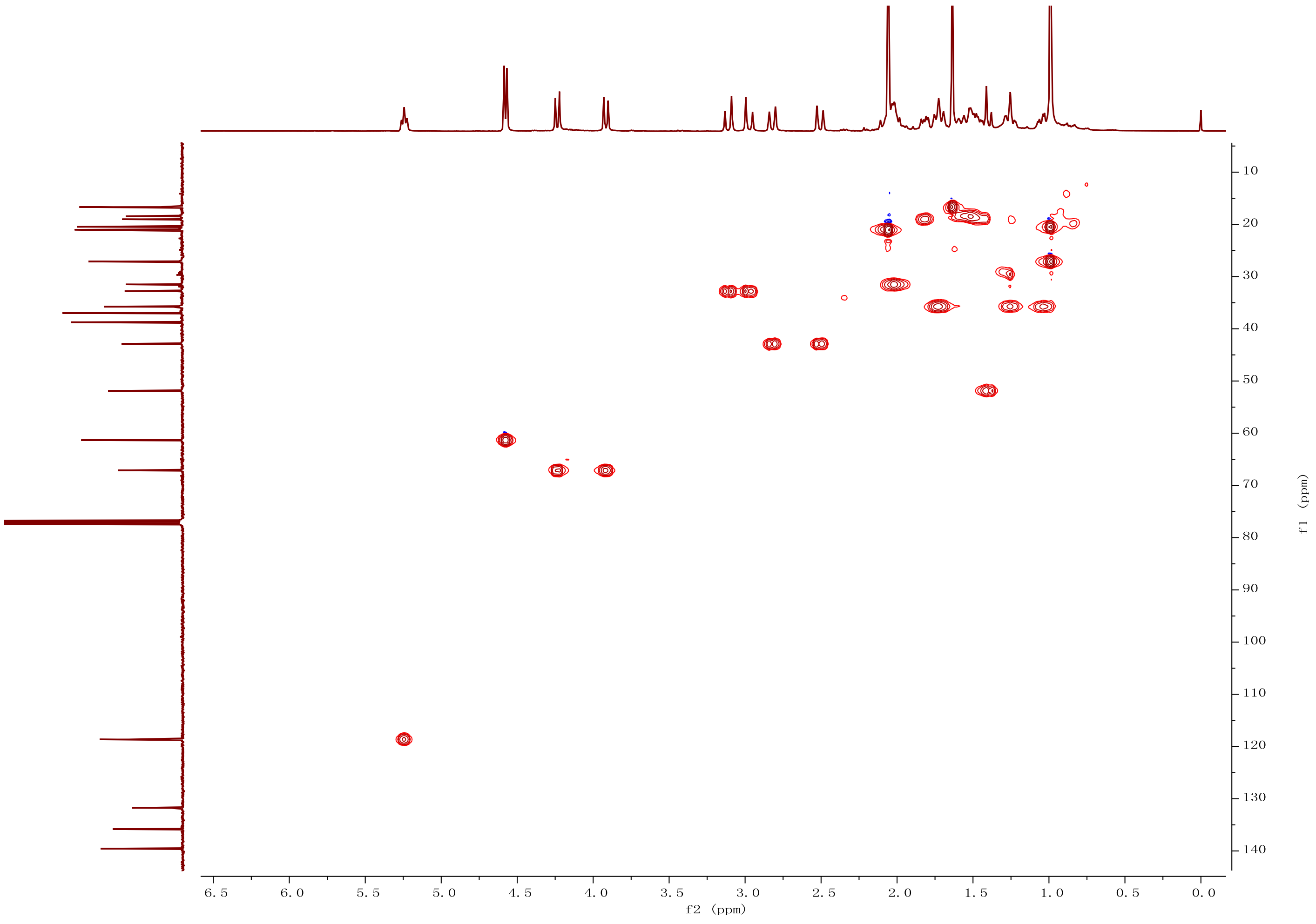

Figure S5. HMBC spectrum of **1** in $CDCl_3$.

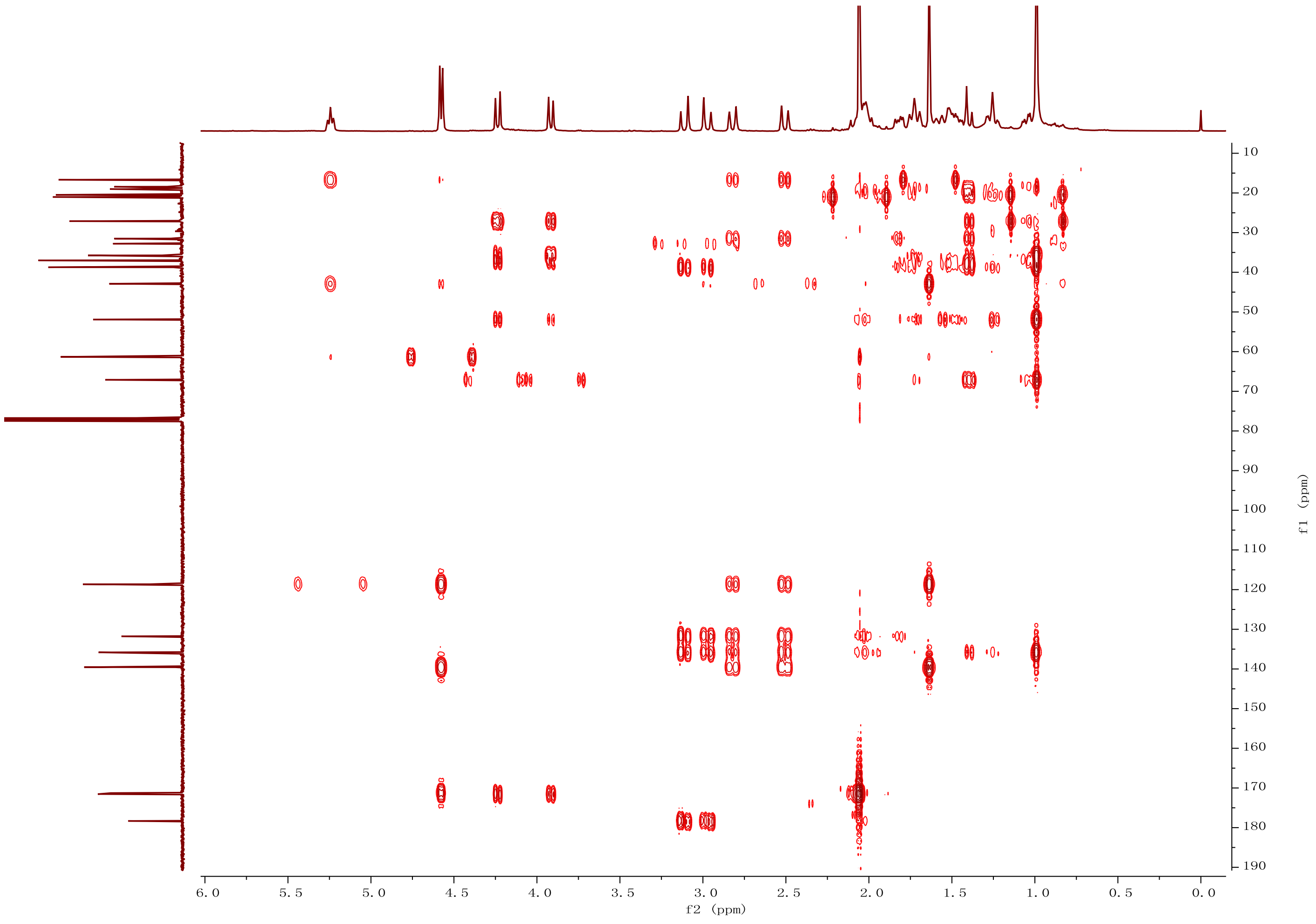

Figure S6. NOESY spectrum of **1** in $CDCl_3$.

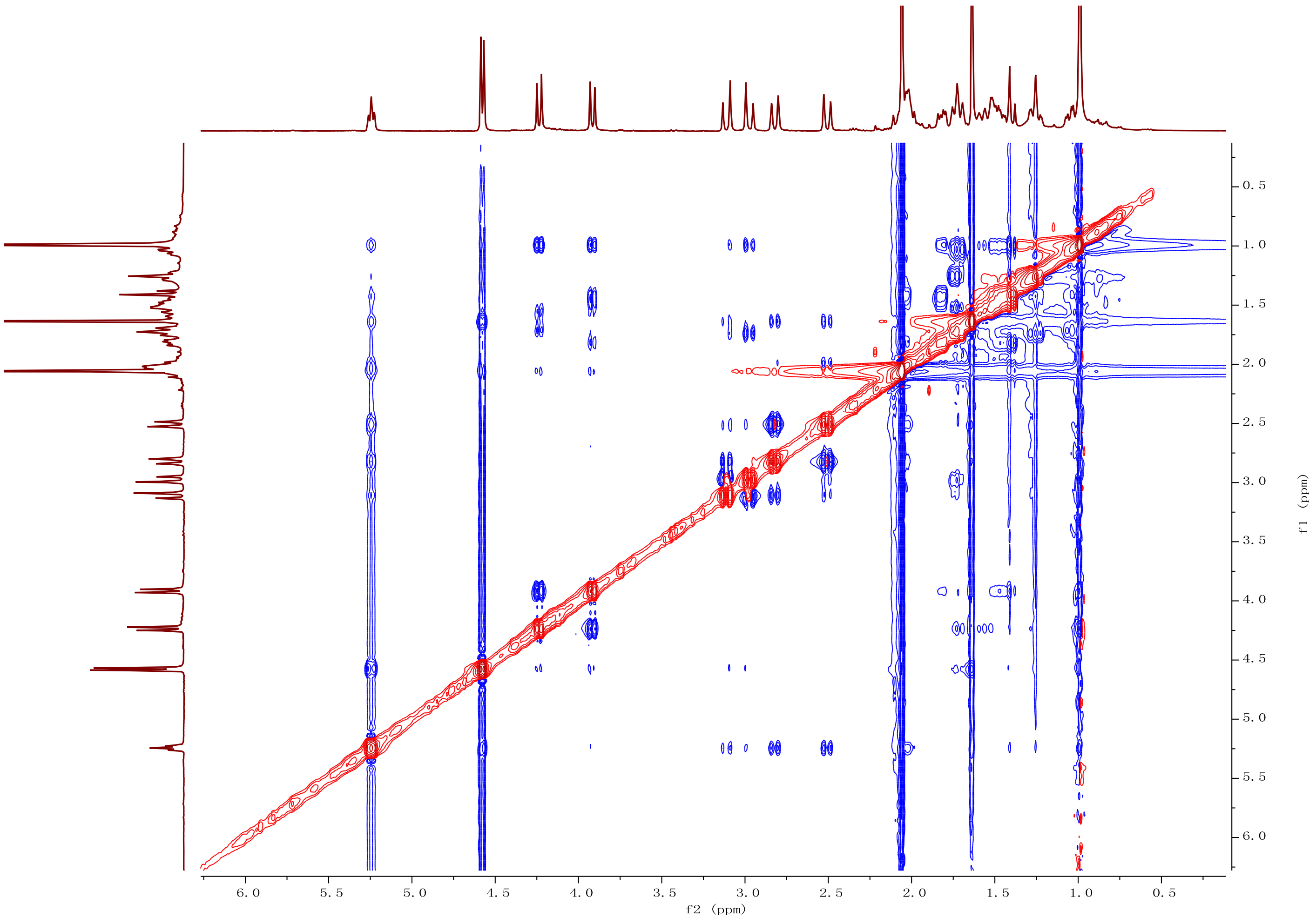

Figure S7. ^{1}H-NMR spectrum of **2** in CD_3OD.

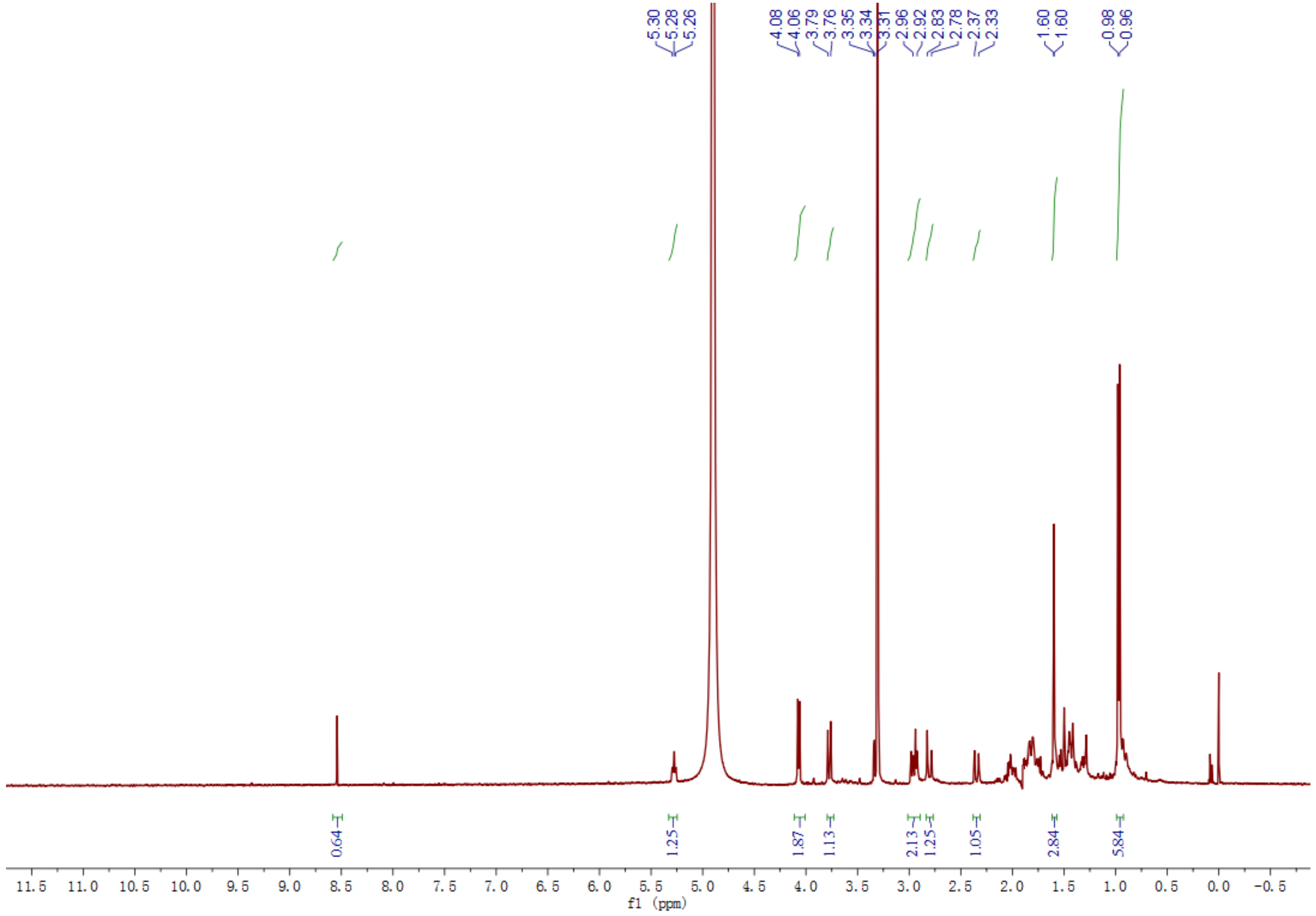

Figure S8. ^{13}C-NMR spectrum of **2** in CD_3OD.

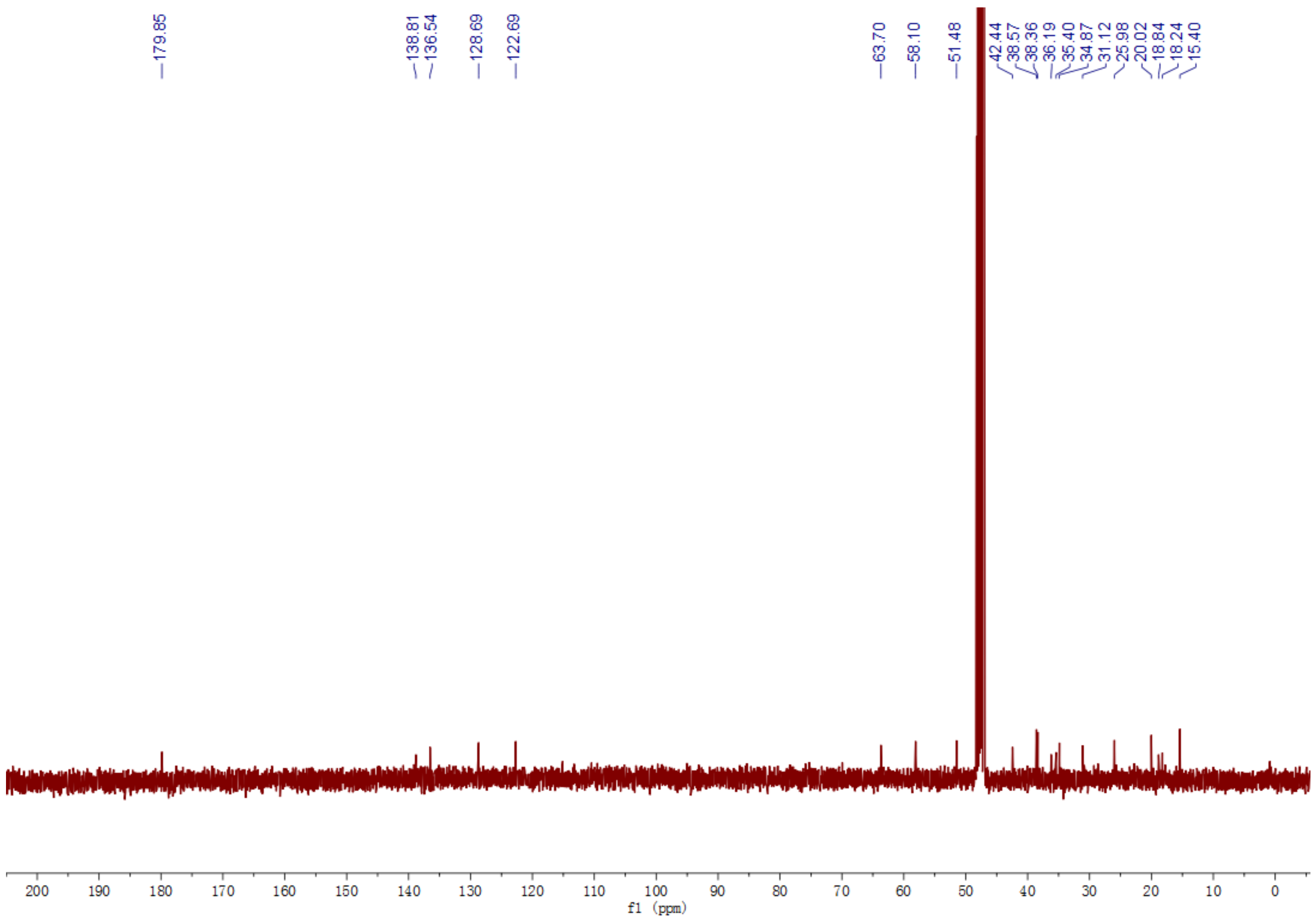

Figure S9. ^{1}H, ^{1}H-COSY spectrum of **2** in CD_3OD.

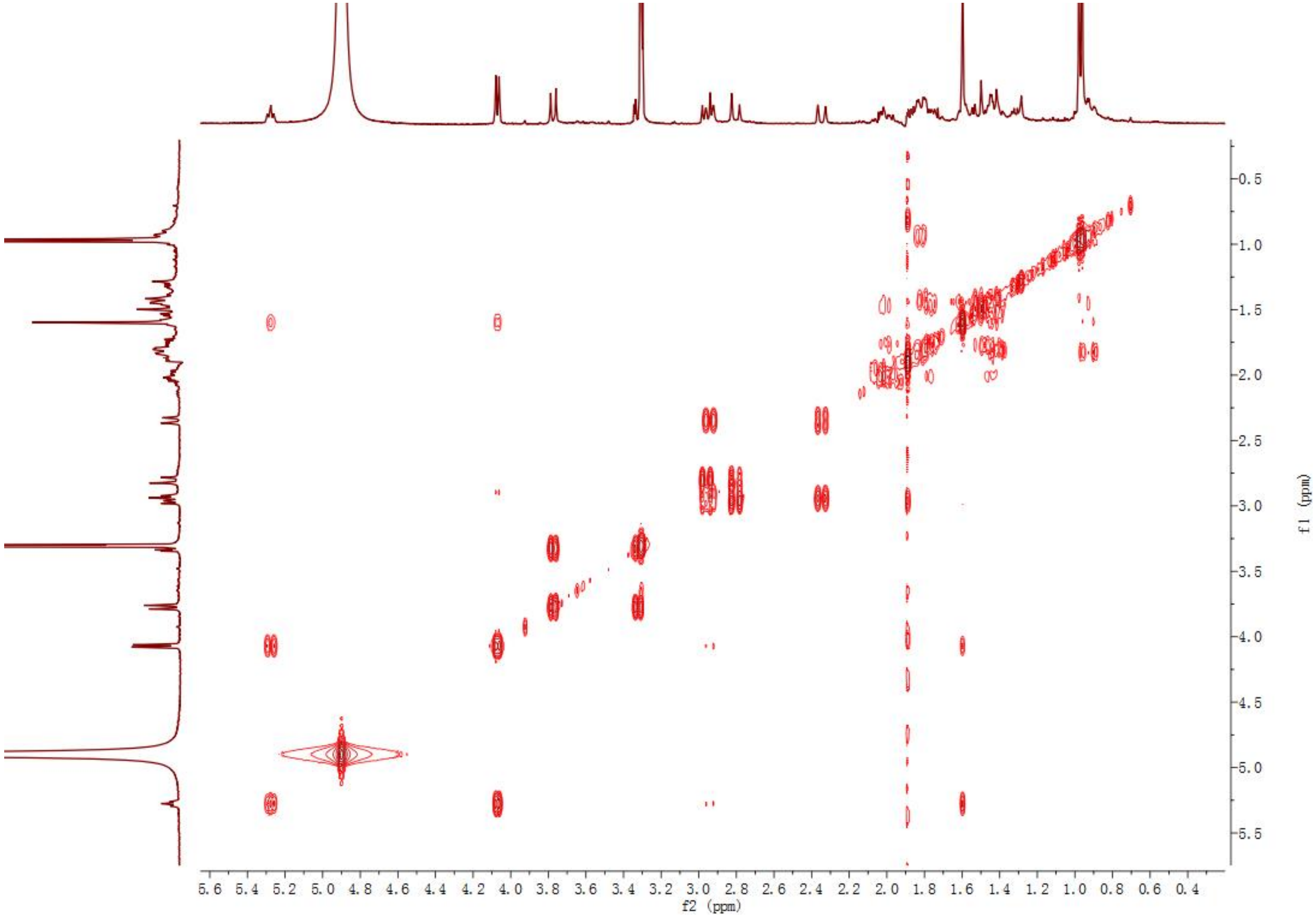

Figure S10. HSQC spectrum of **2** in CD_3OD.

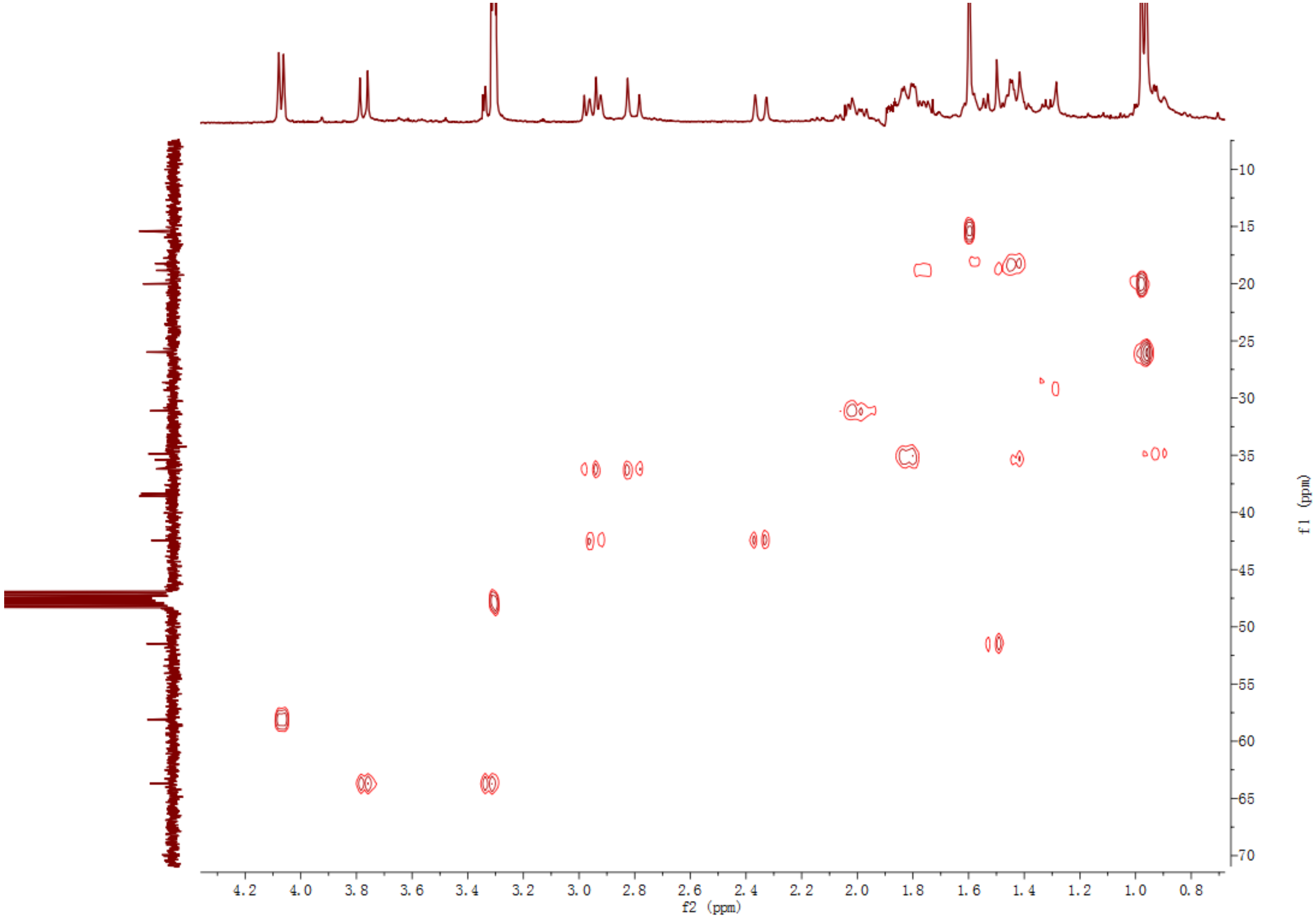

Figure S11. HMBC spectrum of **2** in CD_3OD.

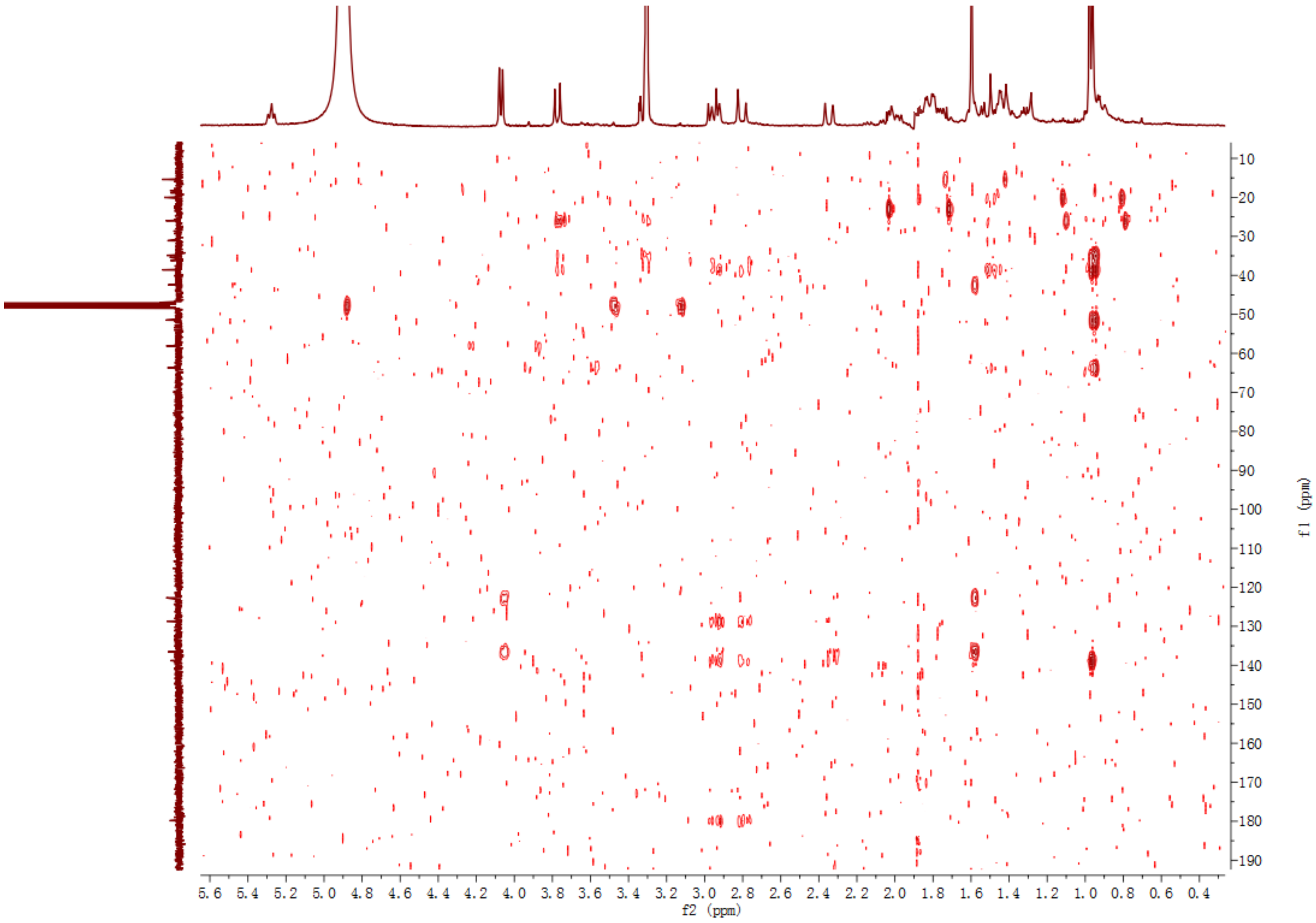

Figure S12. NOESY spectrum of **2** in CD_3OD

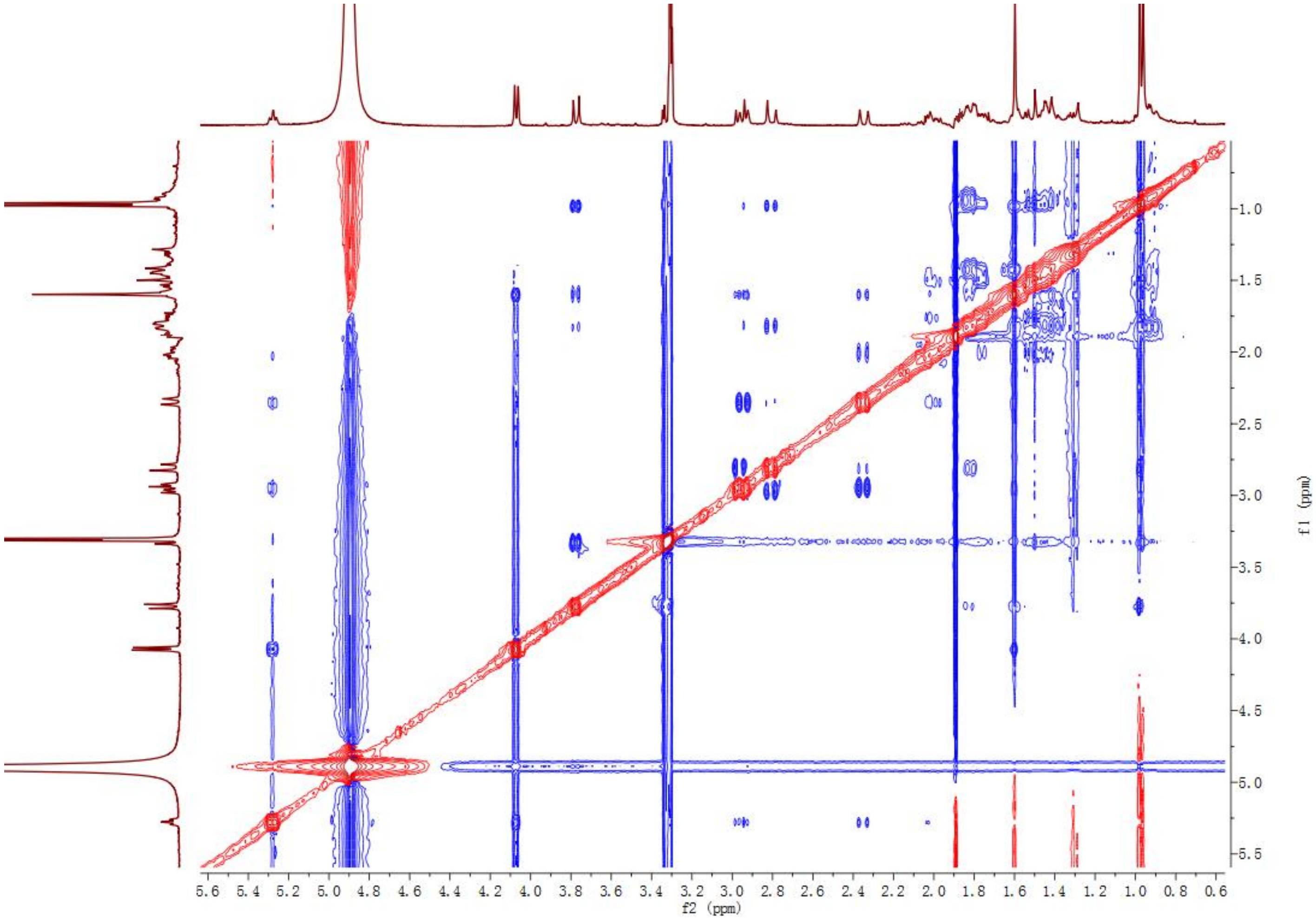

Figure S13. ^{1}H NMR spectrum of **3** in $CDCl_3$.

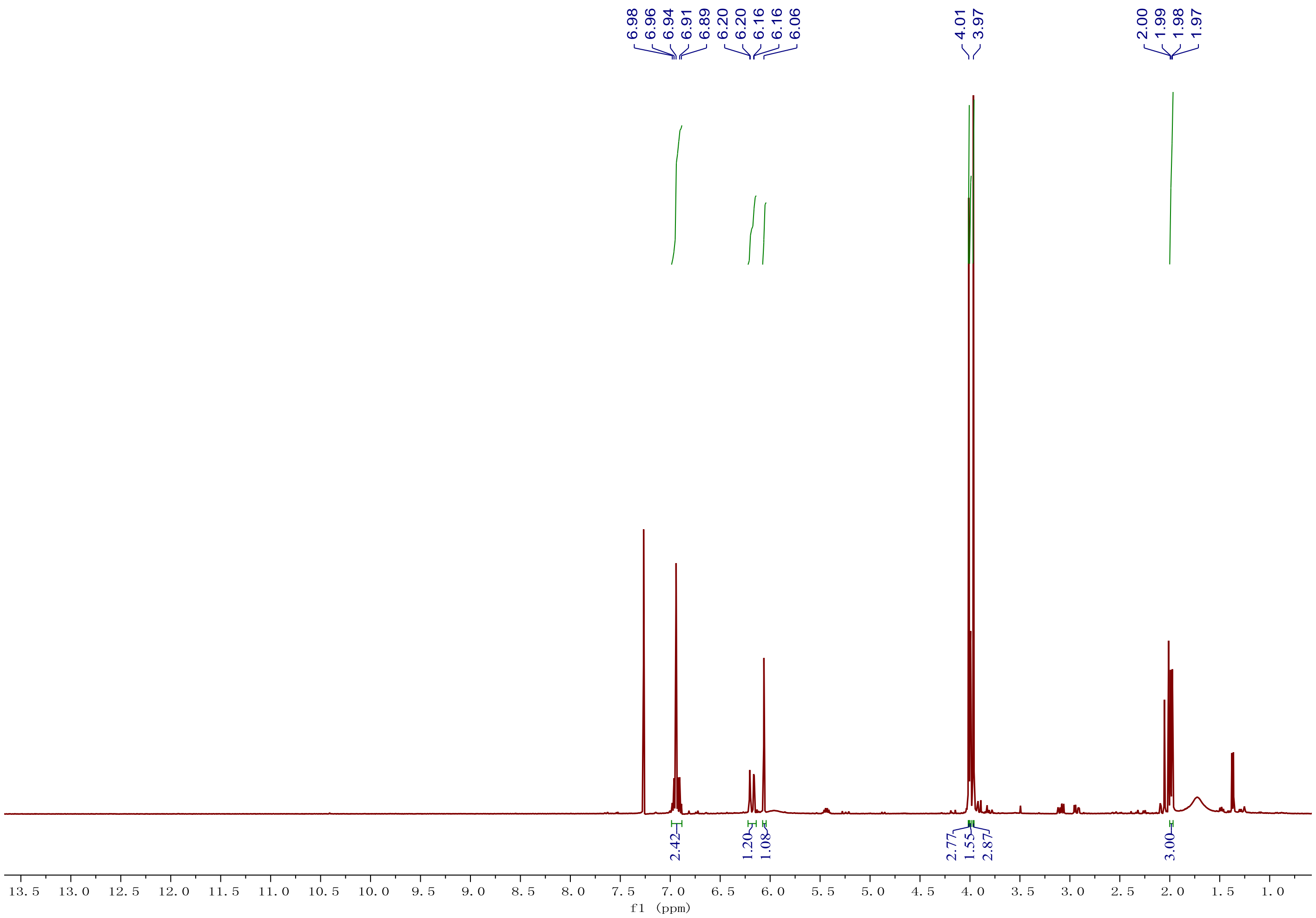

Figure S14. ^{13}C NMR spectrum of **3** in $CDCl_3$.

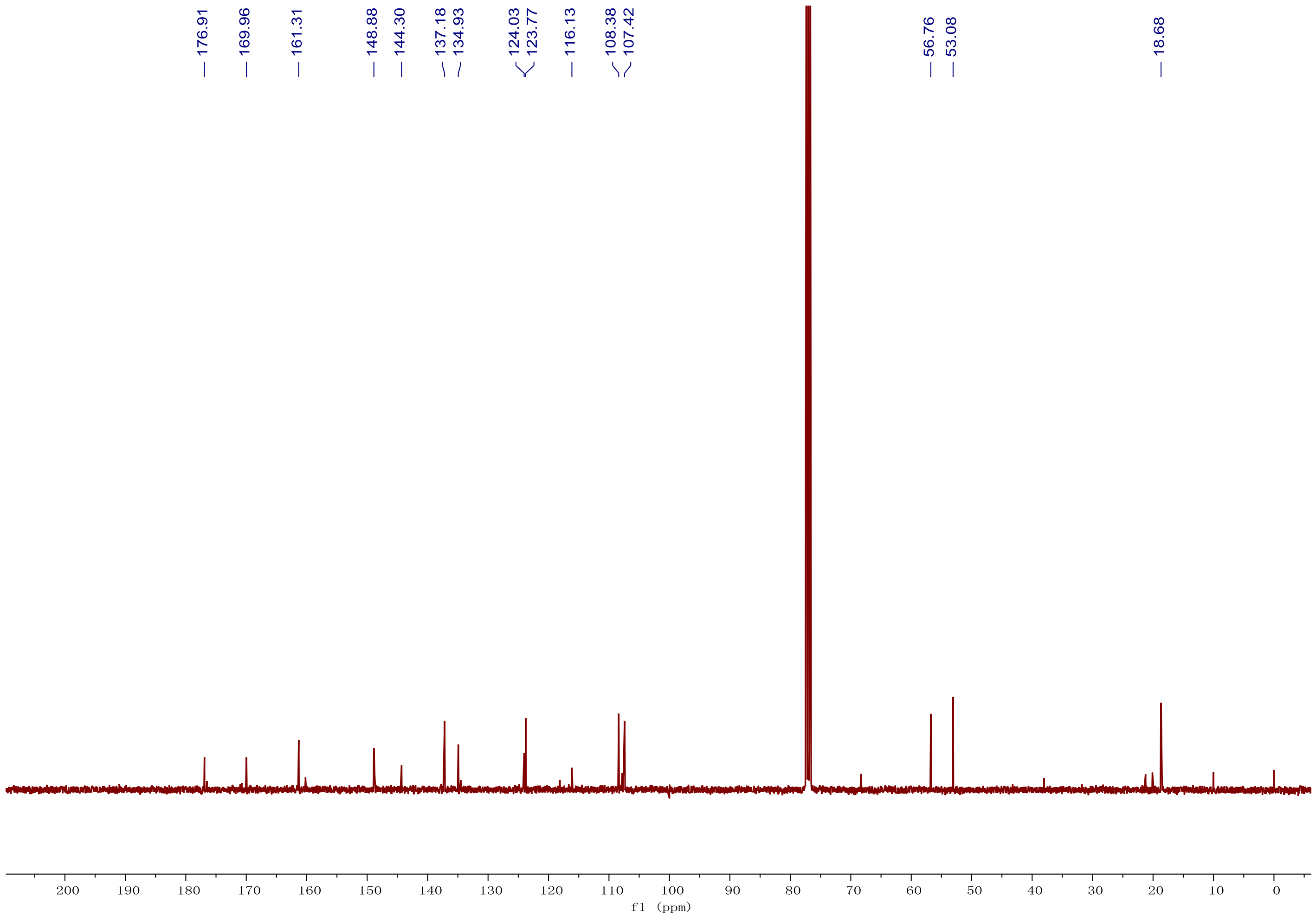

Figure S15. ^{1}H-^{1}H COSY spectrum of **3** in $CDCl_3$.

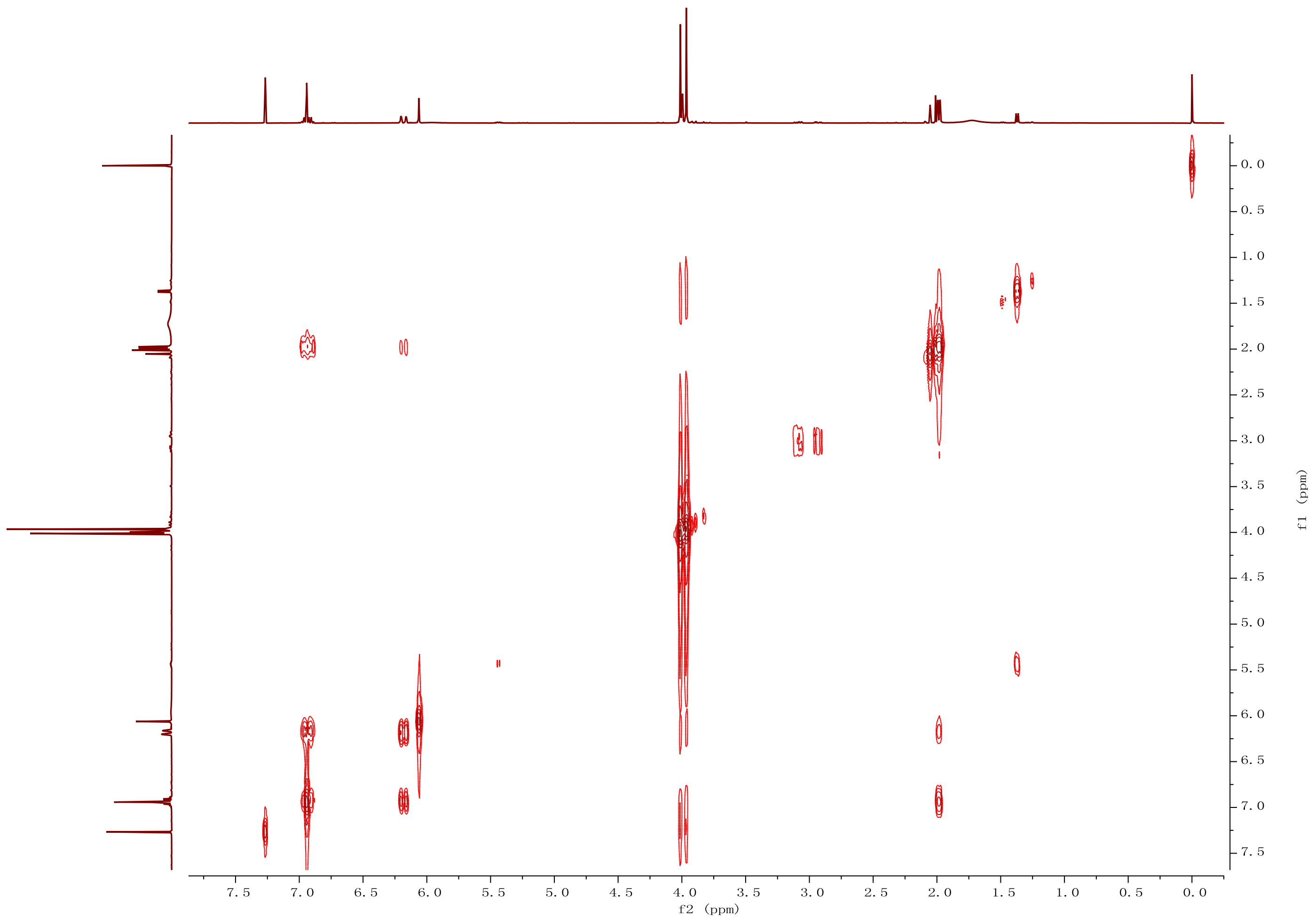

Figure S16. HSQC spectrum of **3** in $CDCl_3$.

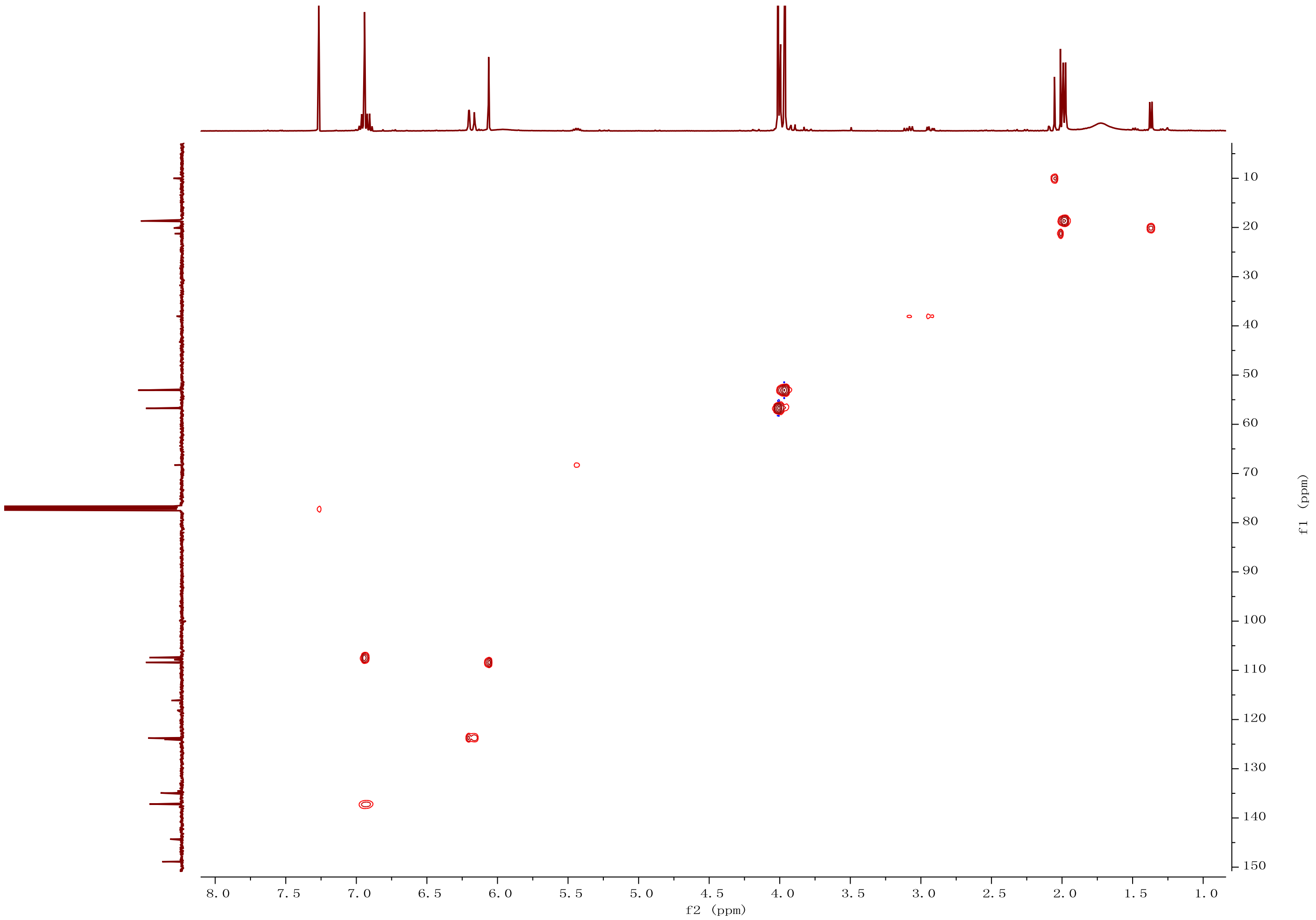

Figure S17. HMBC spectrum of **3** in $CDCl_3$.

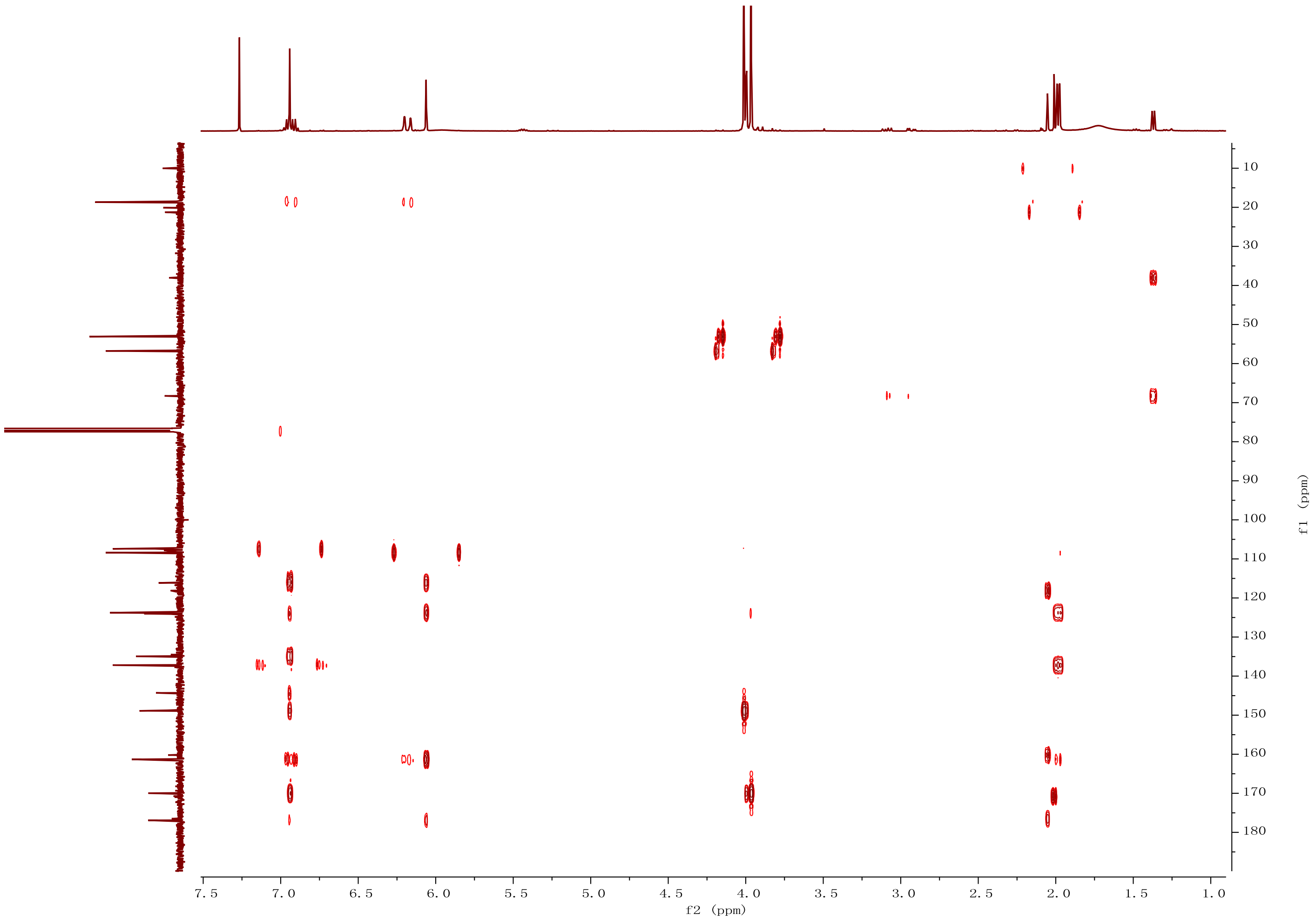

Figure S18. ^{1}H NMR spectrum of **4** in CD_3OD.

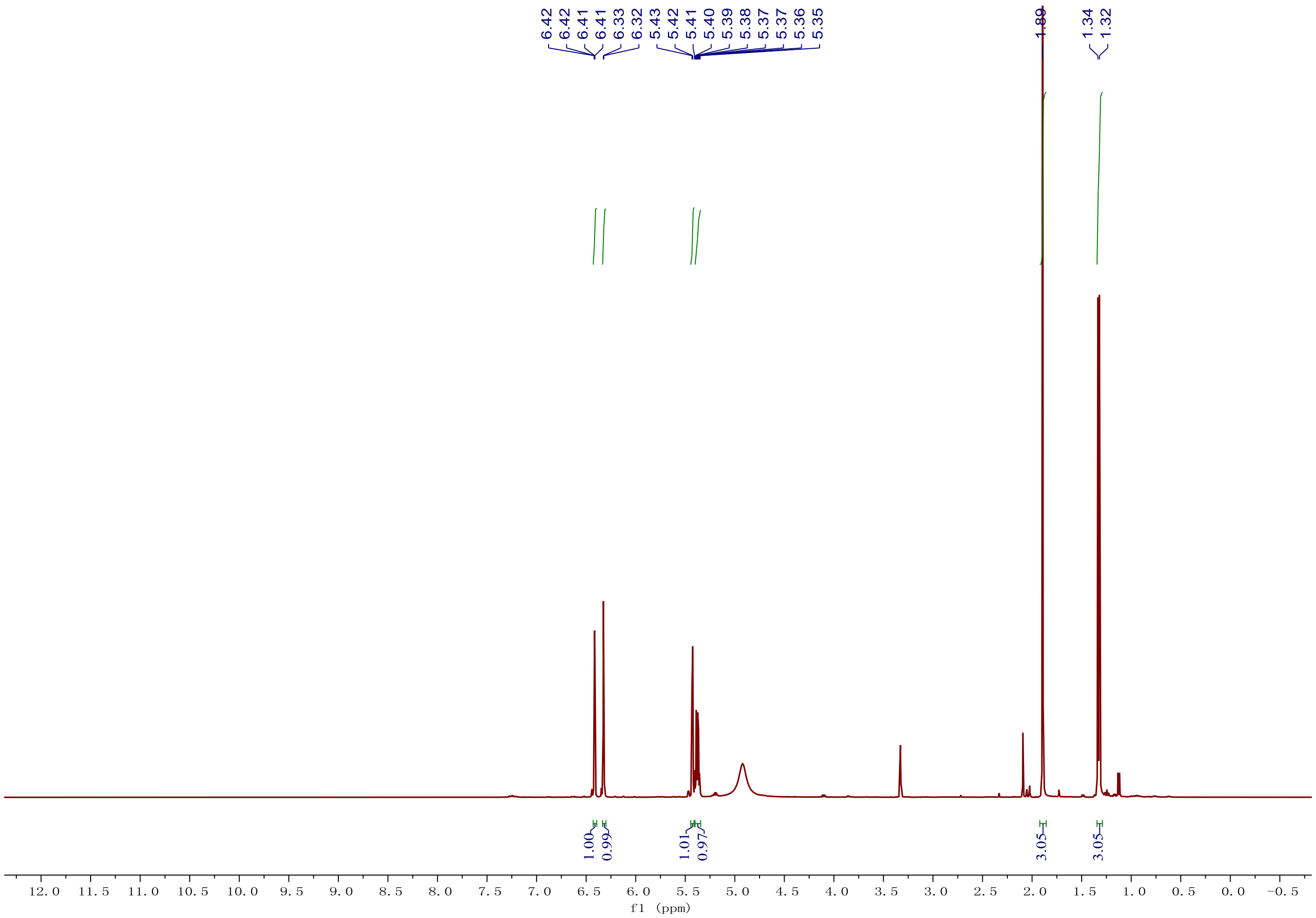

Figure S19. ^{13}C NMR spectrum of **4** in CD_3OD.

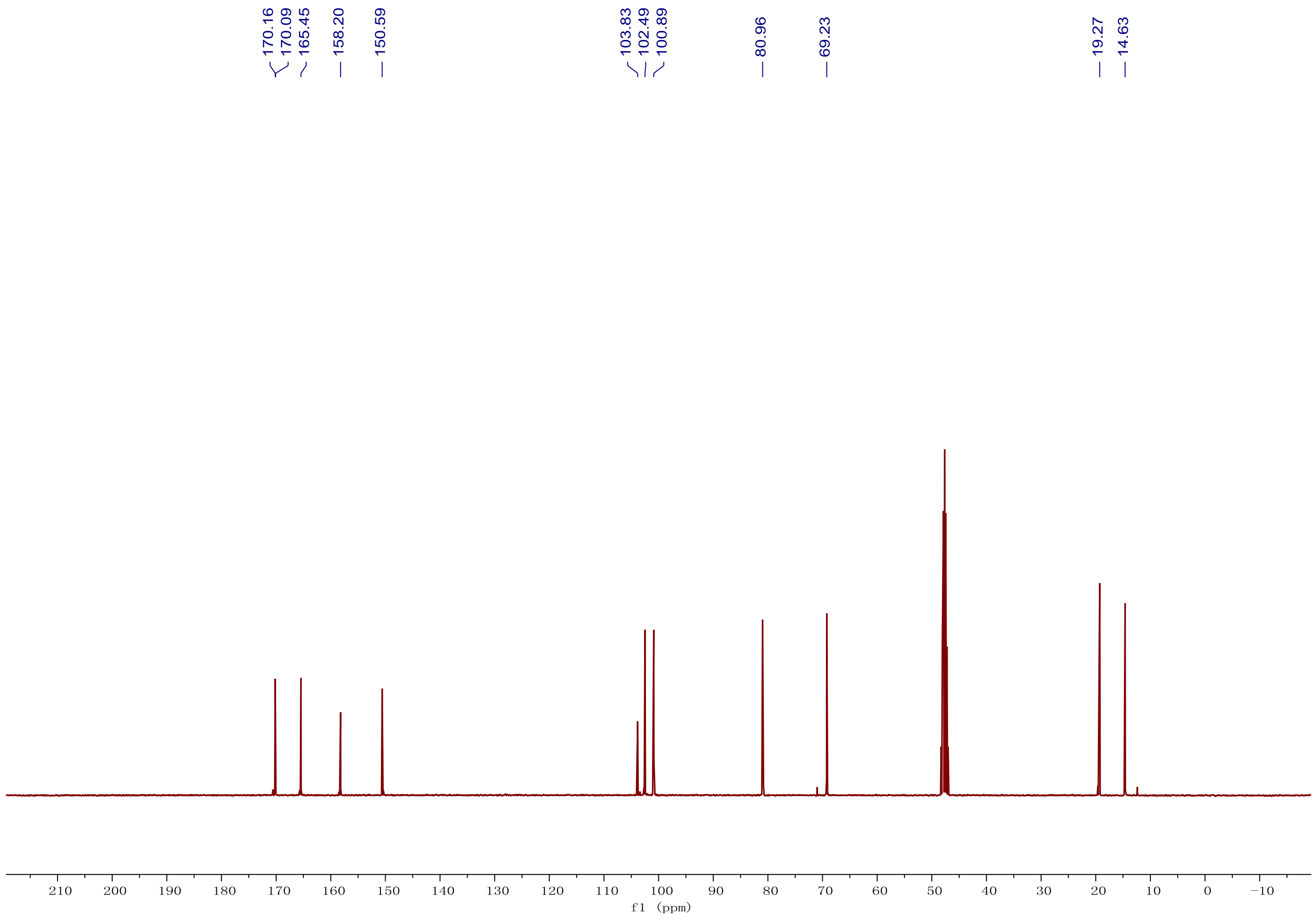

Figure S20. ^1H-^1H COSY spectrum of **4** in CD_3OD.

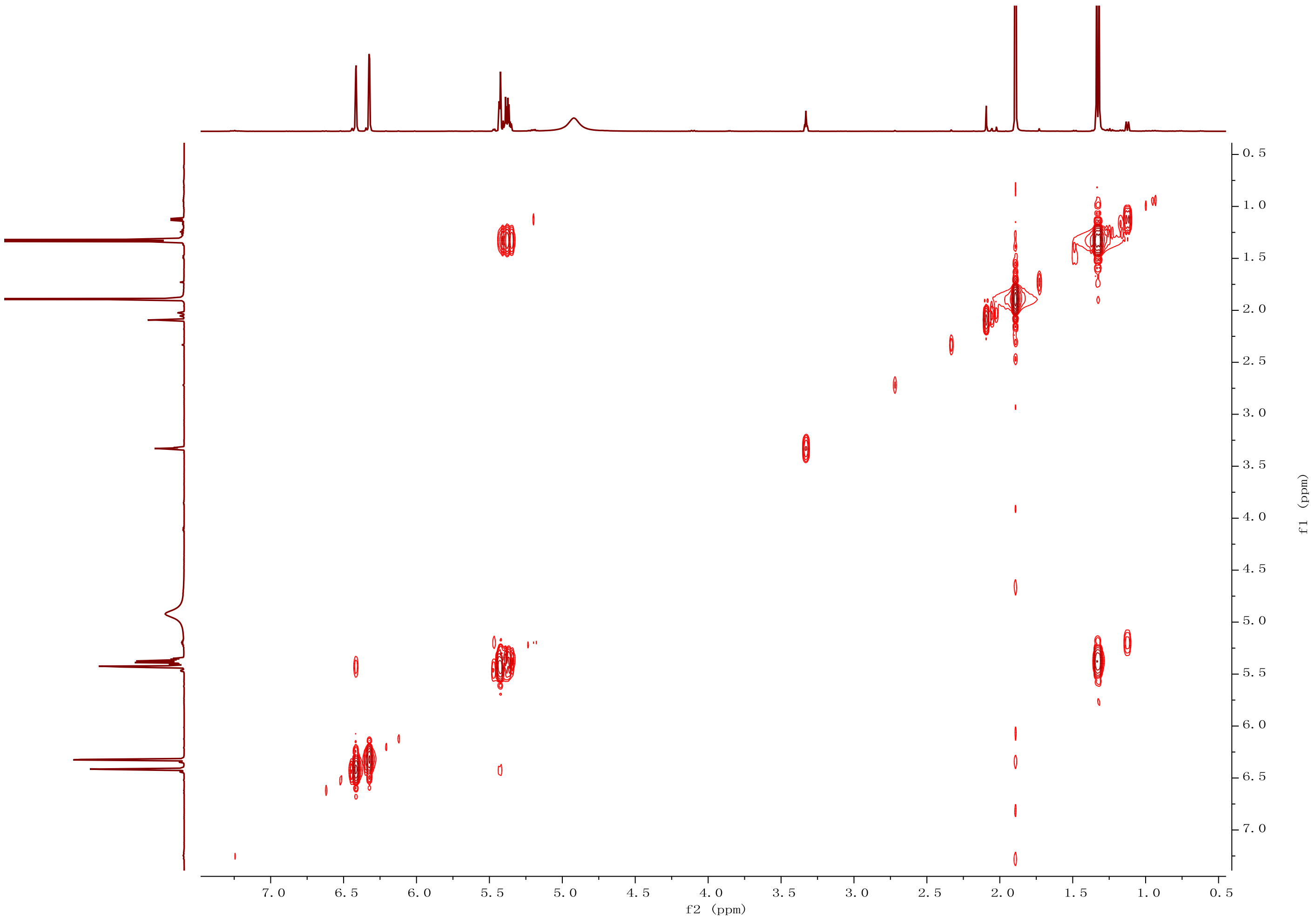

Figure S21. HSQC spectrum of **4** in CD_3OD.

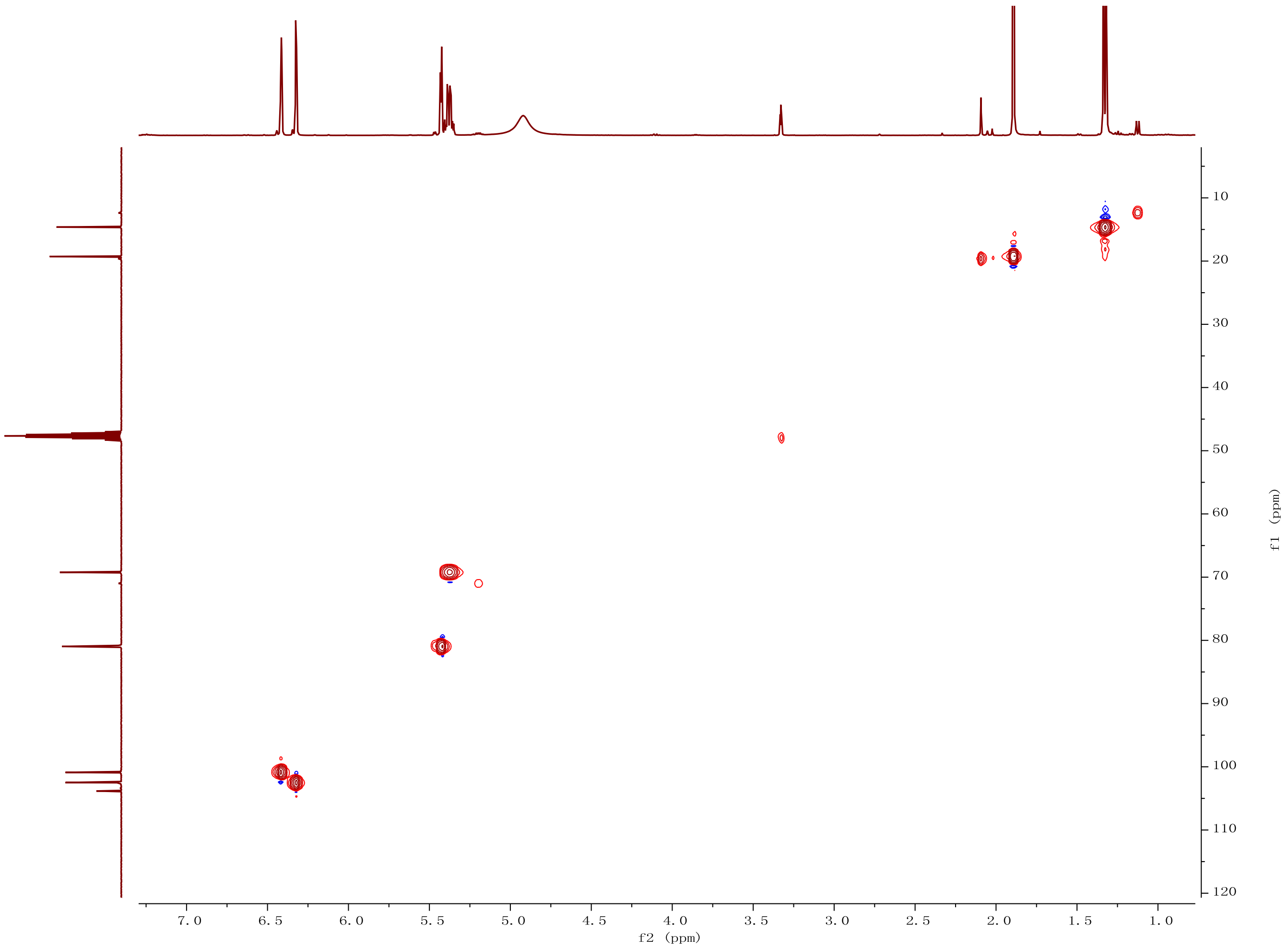

Figure S22. HMBC spectrum of **4** in CD_3OD.

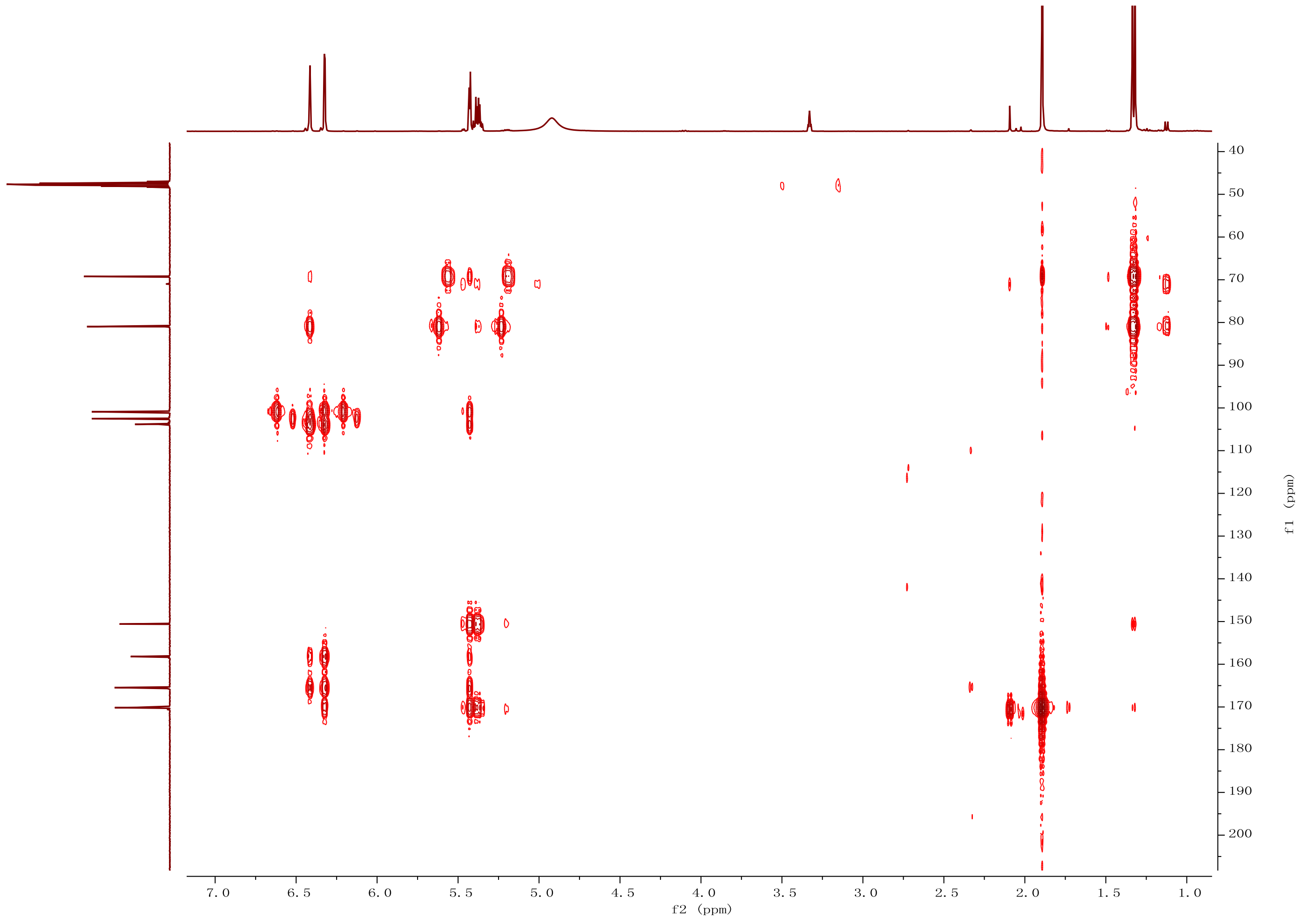

Figure S23. ^{1}H NMR spectrum of **6** in $CDCl_3$

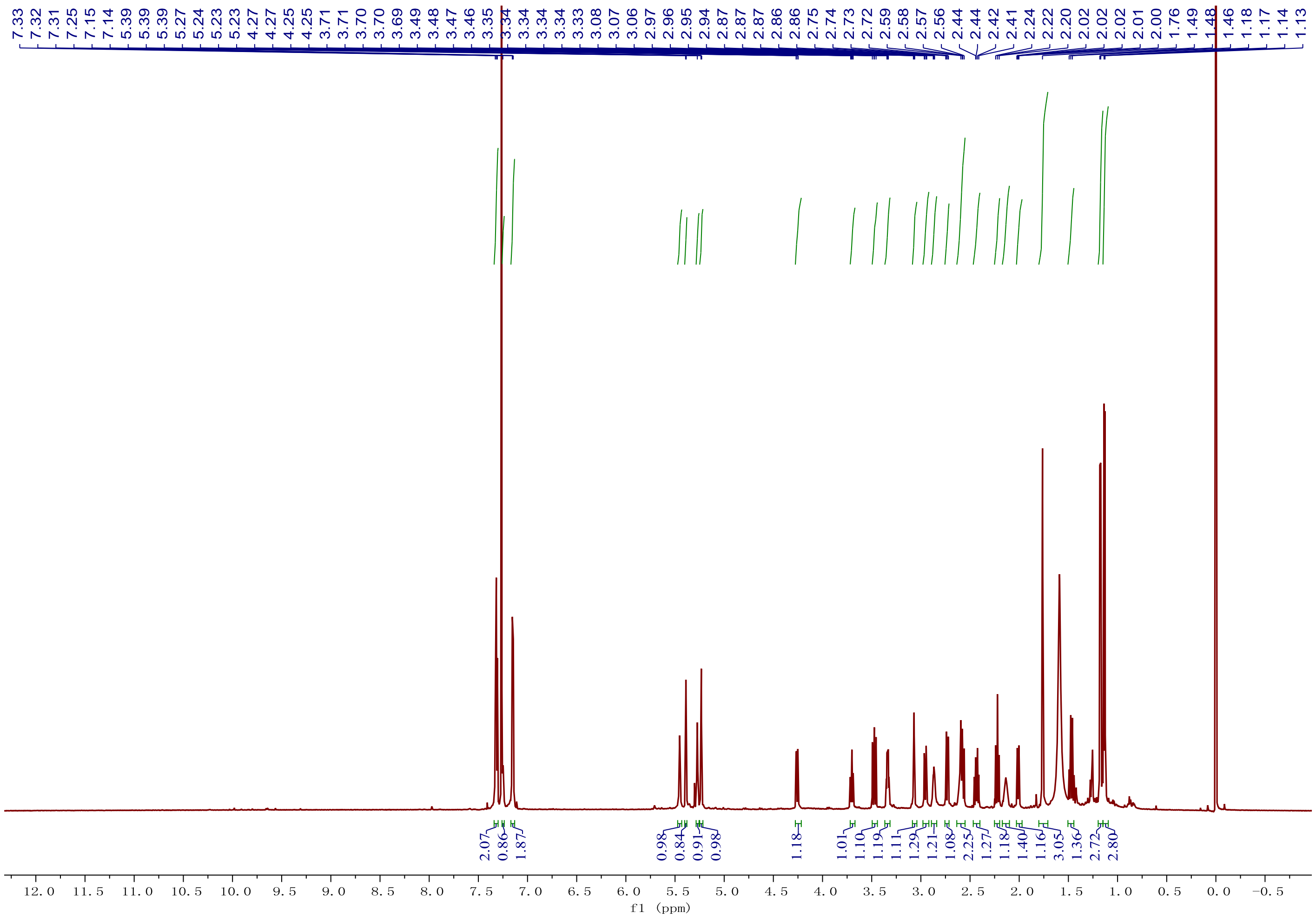

Figure S24. ^{13}C NMR spectrum of **6** in $CDCl_3$.

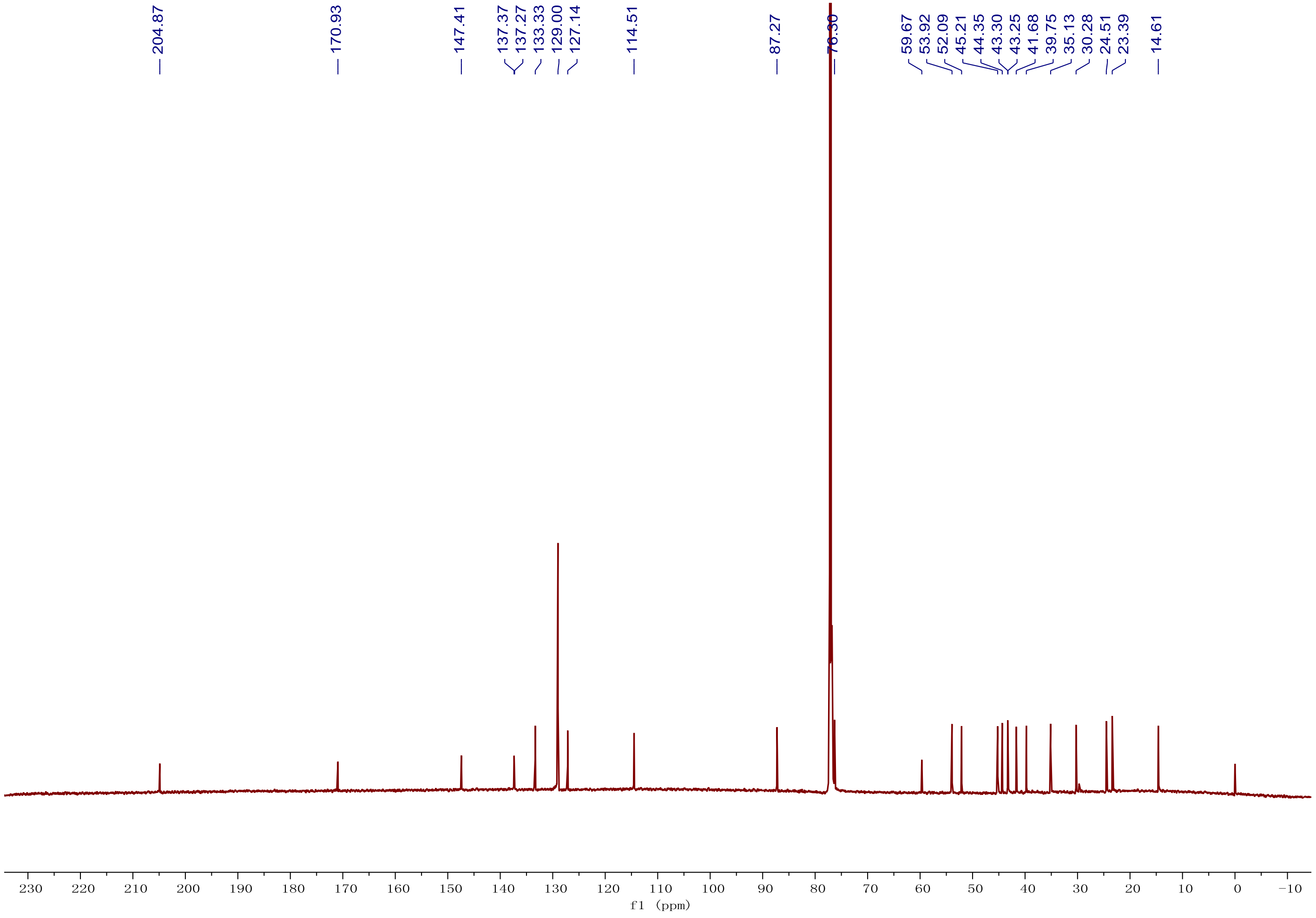

Figure S25. ^{1}H, ^{1}H-COSY spectrum of **6** in $CDCl_3$.

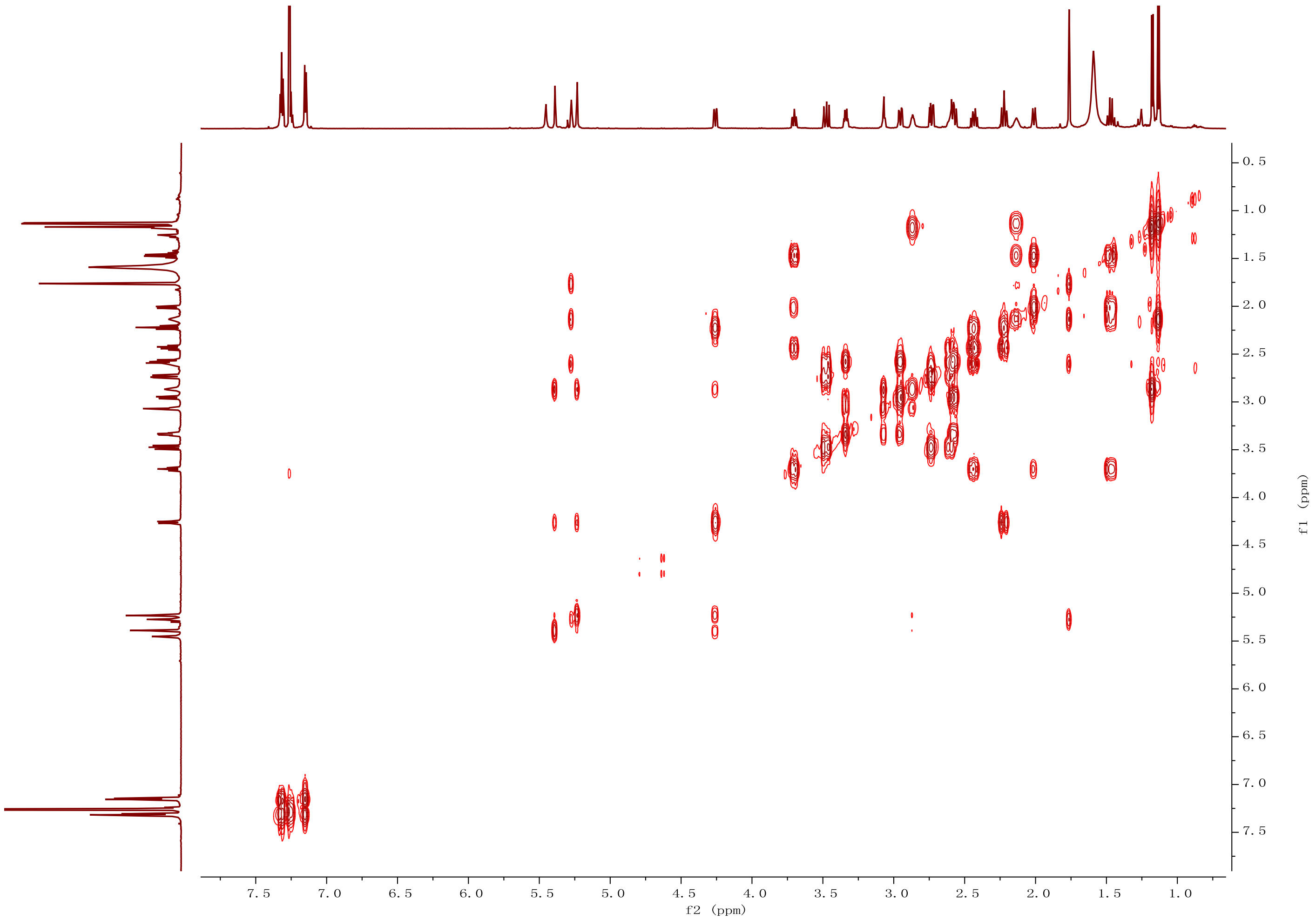

Figure S26. HSQC spectrum of **6** in $CDCl_3$.

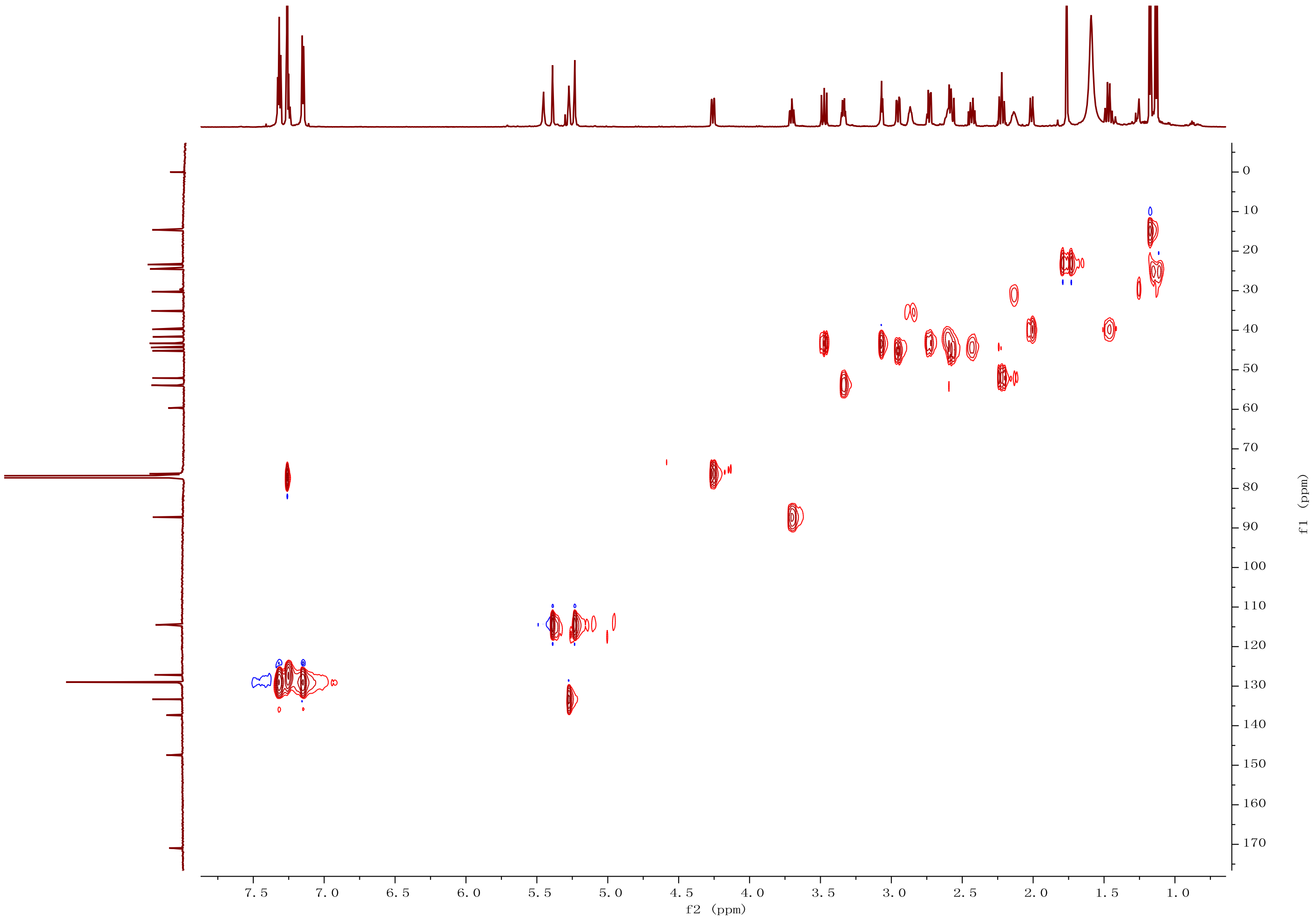

Figure S27. HMBC spectrum of **6** in $CDCl_3$.

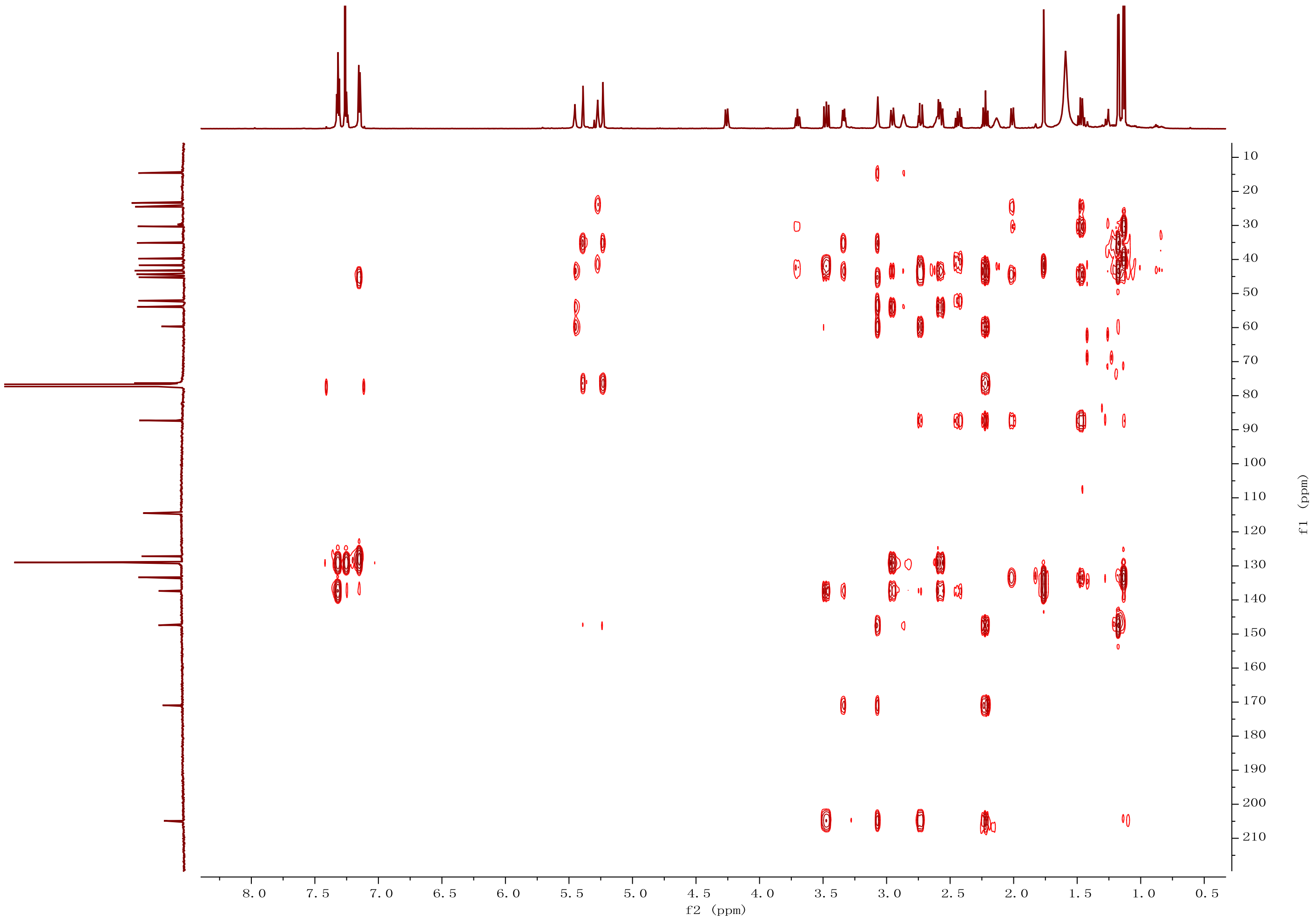

Figure S28. NOESY spectrum of **6** in $CDCl_3$.

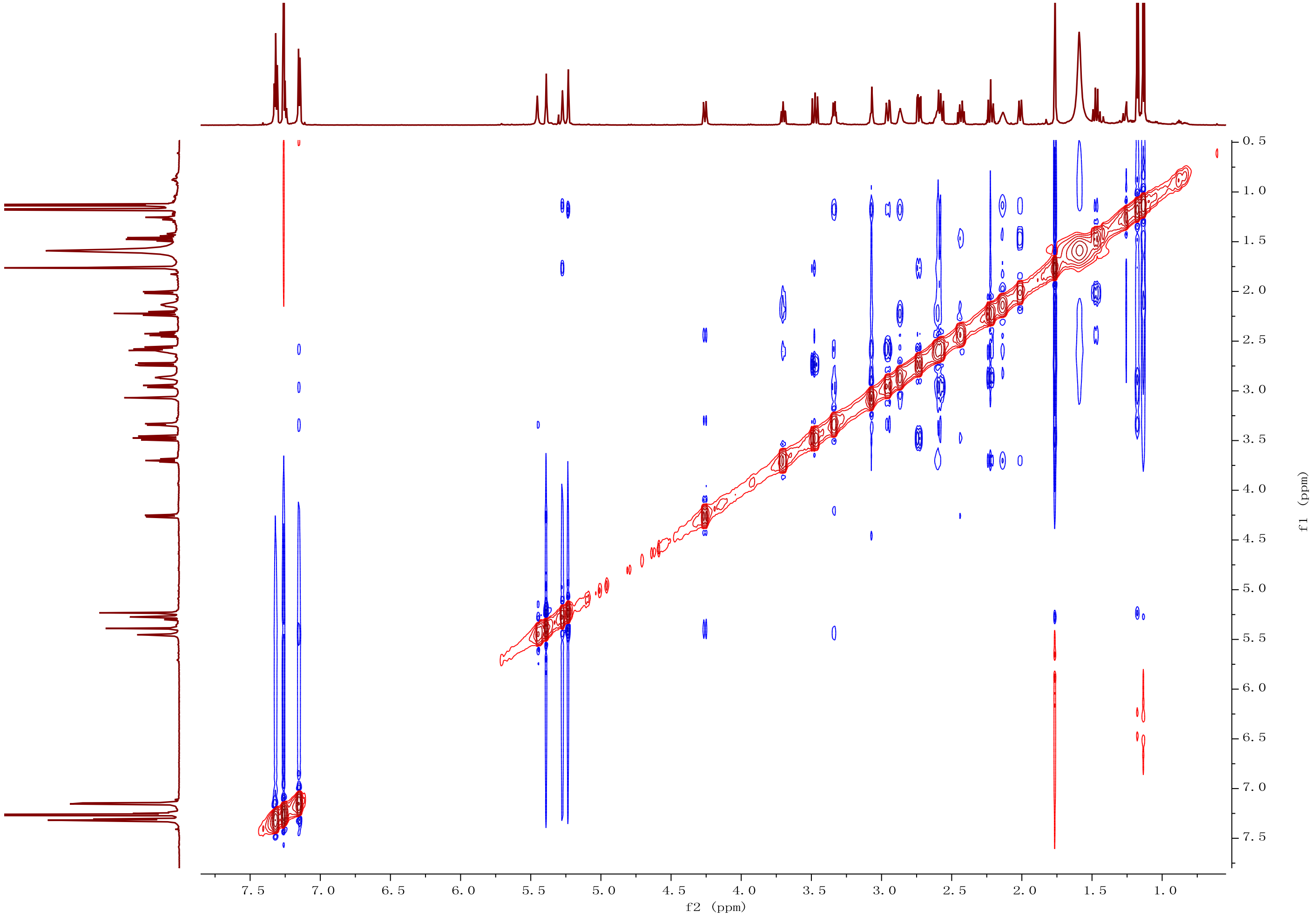

Figure S29. ^{1}H NMR spectrum of **8** in $CDCl_3$.

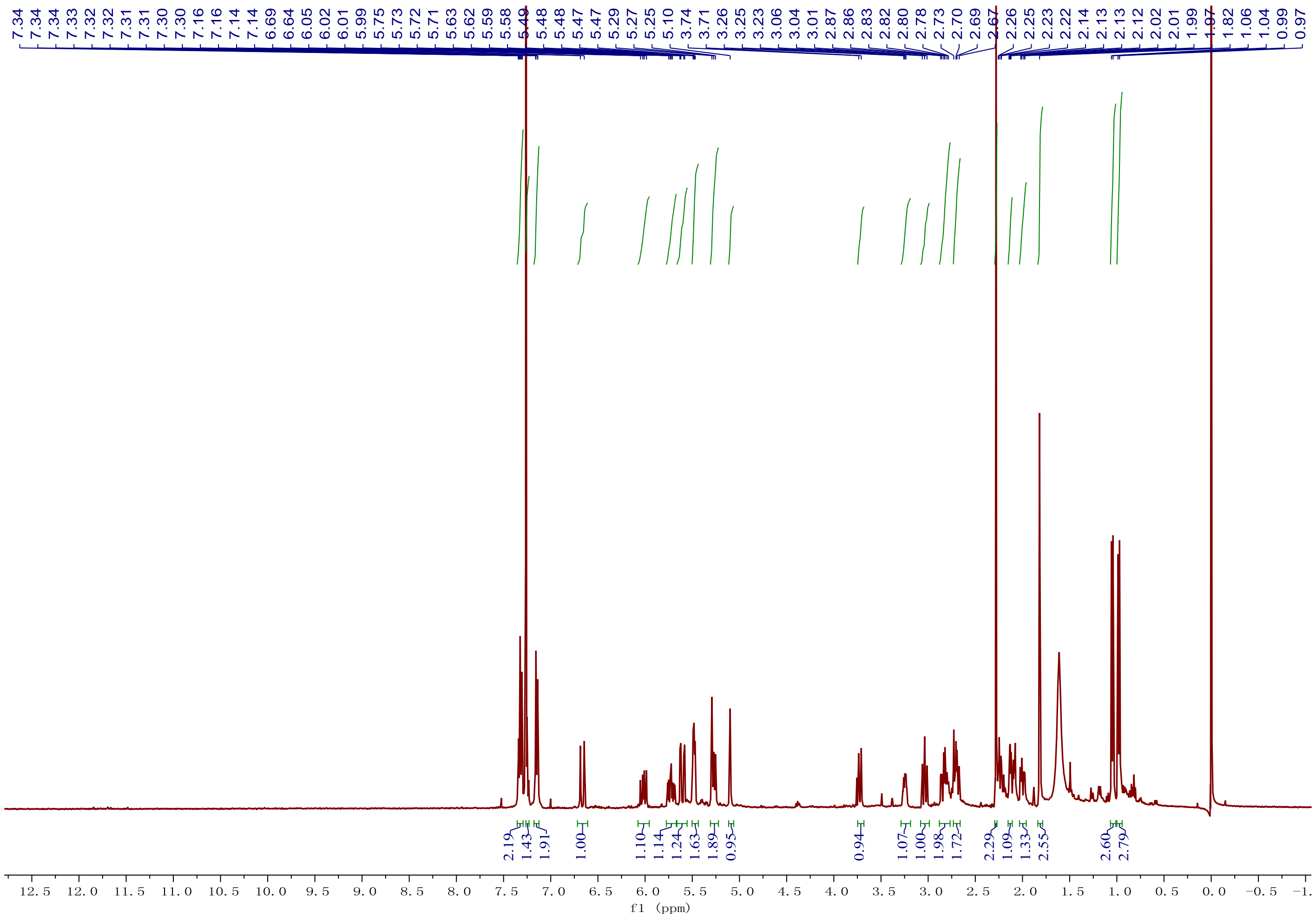

Figure S30. ^{13}C NMR spectrum of **8** in $CDCl_3$.

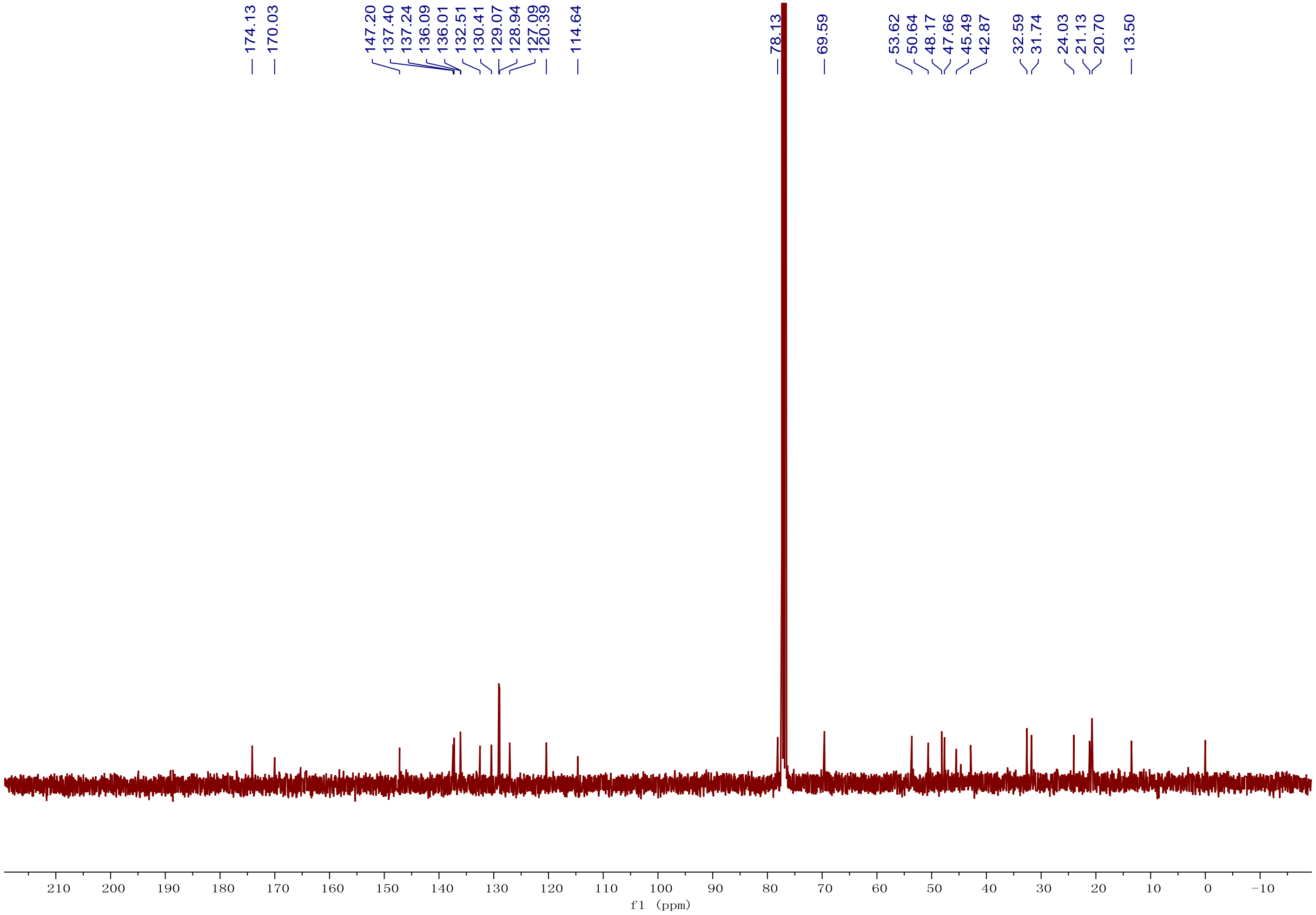

Figure S31. ^{1}H, ^{1}H-COSY spectrum of **8** in $CDCl_3$.

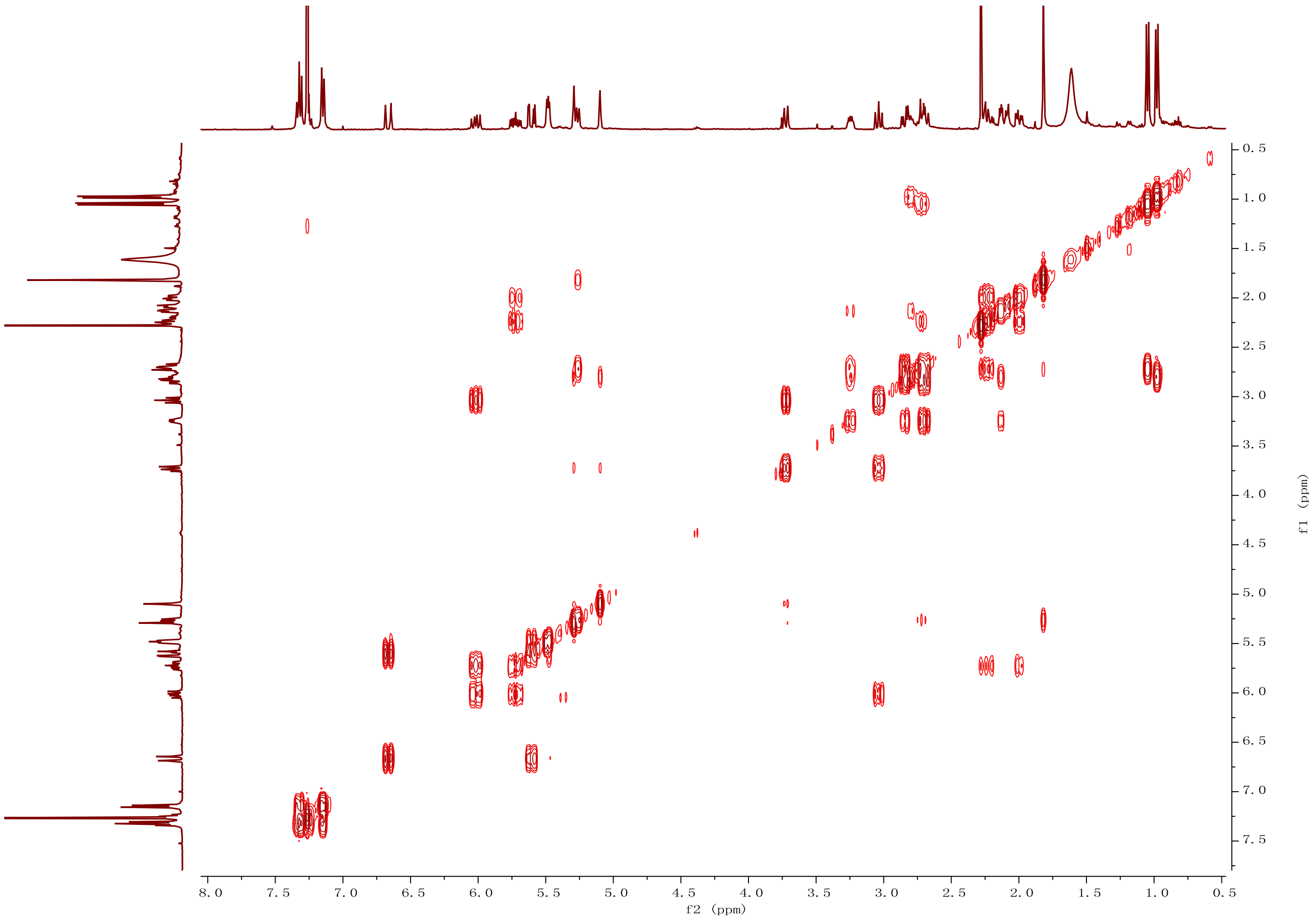

Figure S32. HSQC spectrum of **8** in $CDCl_3$.

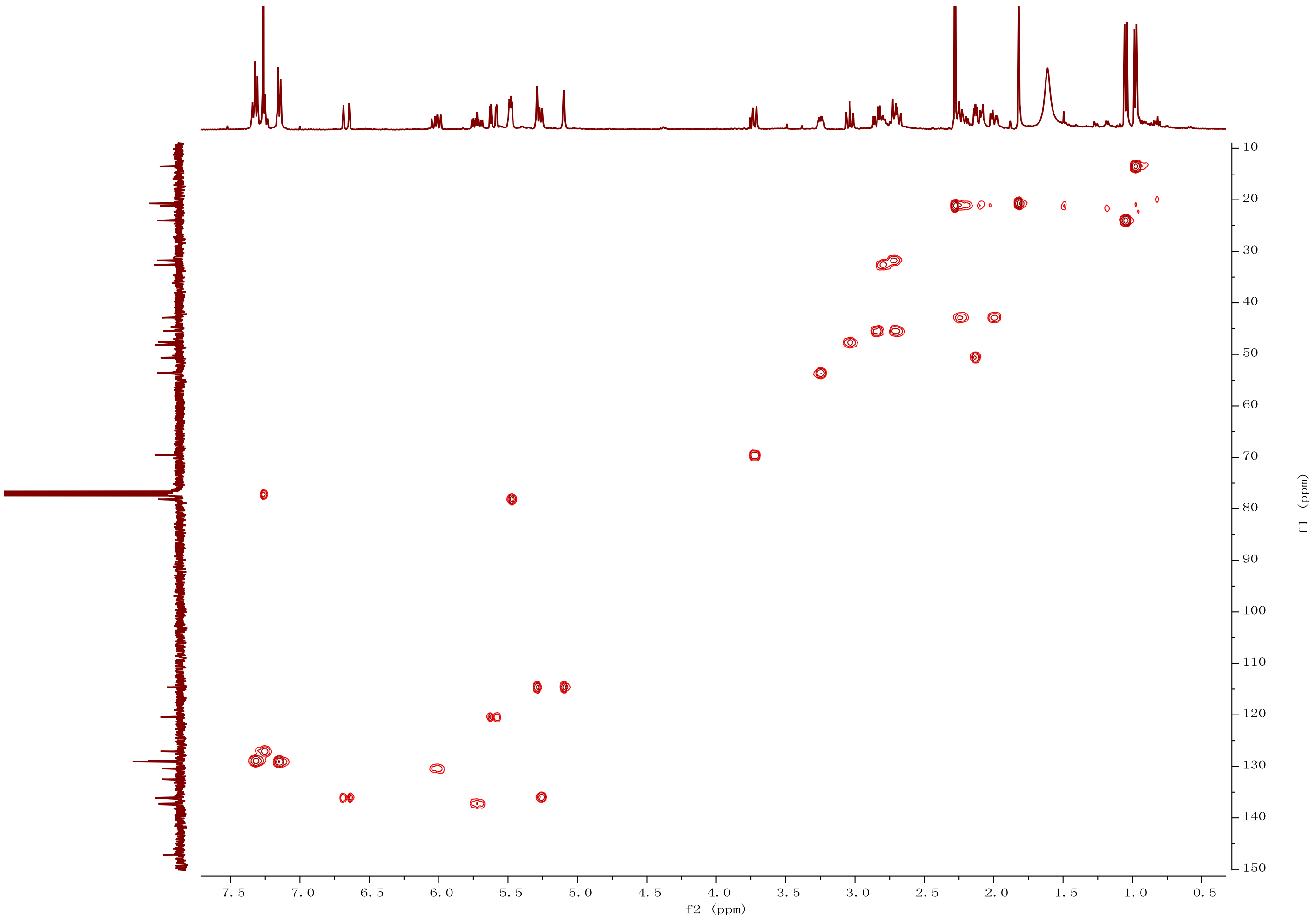

Figure S33. HMBC spectrum of **8** in $CDCl_3$.

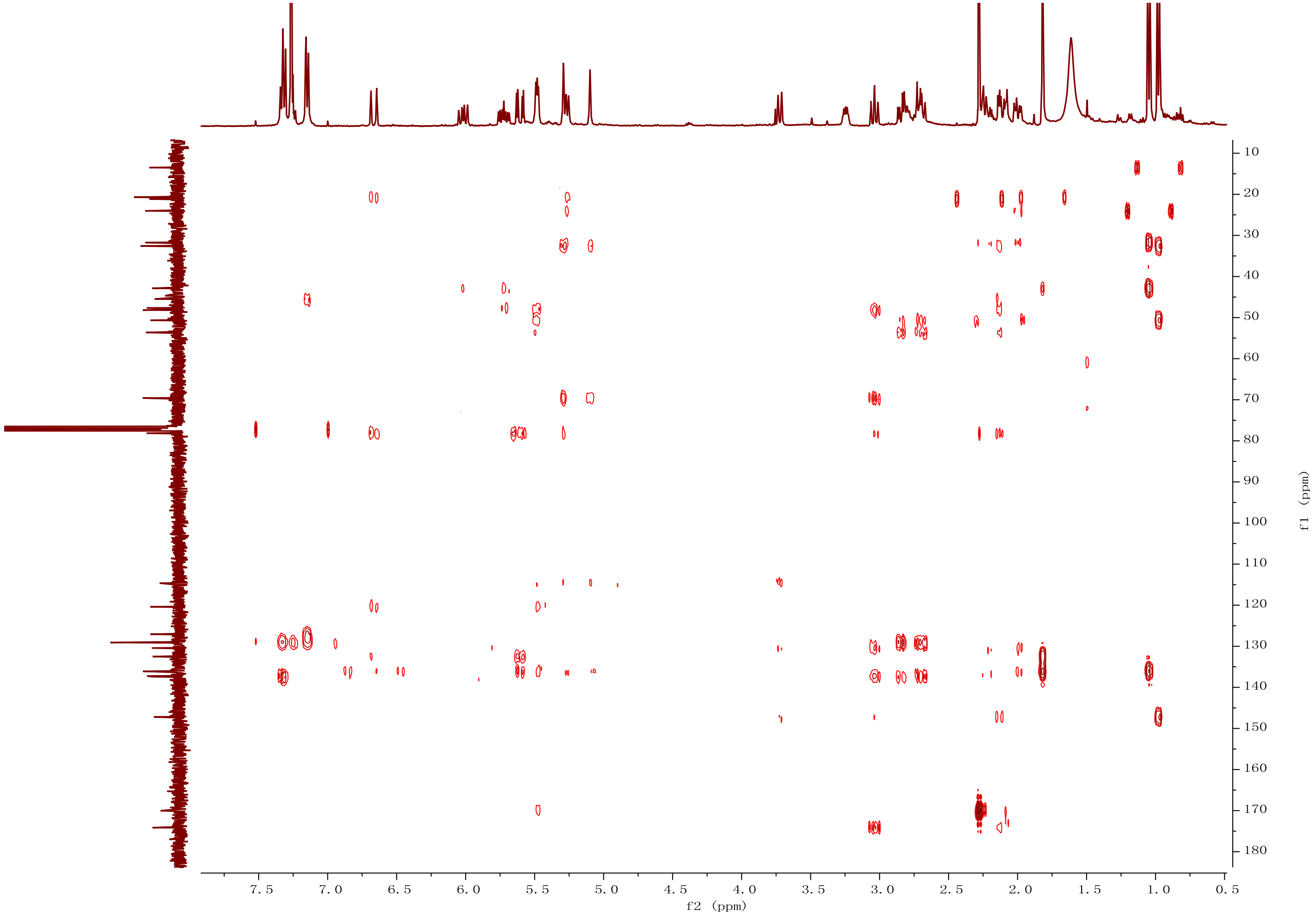

Figure S34. NOESY spectrum of **8** in $CDCl_3$.

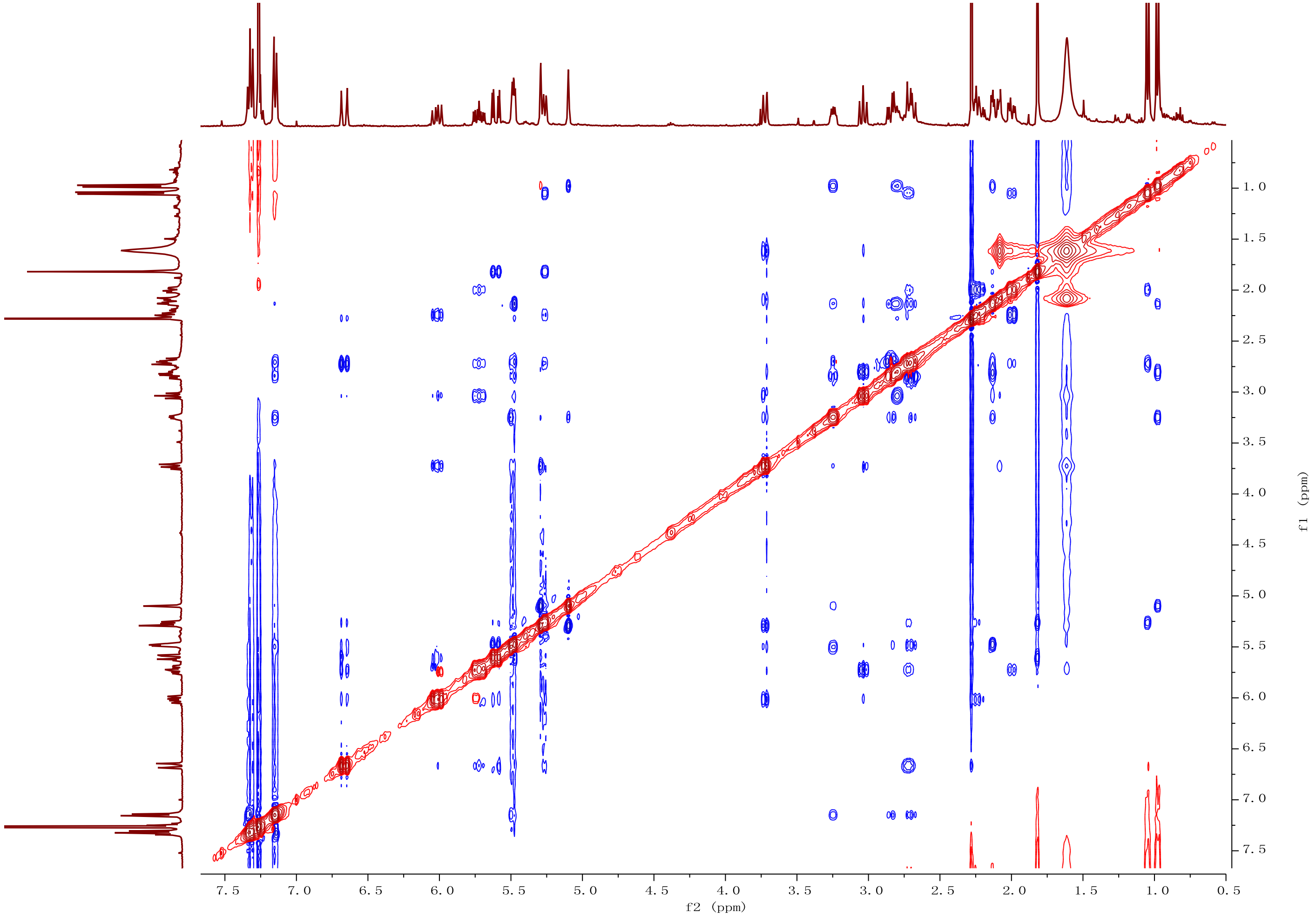

Figure S35. HR-ESI-MS spectrum of **1**.

Acquisition Parameter

Source Type	ESI	Ion Polarity	Positive	Set Nebulizer	0.4 Bar
Focus	Active	Set Capillary	4500 V	Set Dry Heater	180 °C
Scan Begin	70 m/z	Set End Plate Offset	-500 V	Set Dry Gas	4.0 l/min
Scan End	1500 m/z	Set Charging Voltage	0 V	Set Divert Valve	Waste
		Set Corona	0 nA	Set APCI Heater	0 °C

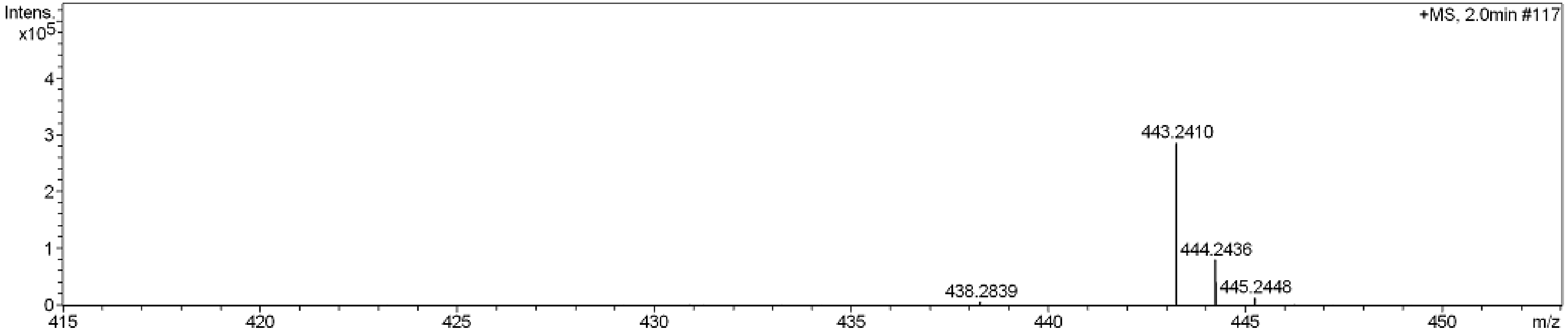

Meas. m/z	#	Ion Formula	Score	m/z	err [ppm]	err [mDa]	mSigma	rdb	e⁻ Conf	N-Rule
443.2410	1	C24H36NaO6	100.00	443.2404	1.3	0.6	7.8	6.5	even	ok
863.4917	1	C48H72NaO12	100.00	863.4916	0.1	0.1	27.7	12.5	even	ok

Figure S36. HR-ESI-MS spectrum of **2**.

Acquisition Parameter

Source Type	ESI	Ion Polarity	Positive	Set Nebulizer	0.4 Bar
Focus	Active	Set Capillary	4500 V	Set Dry Heater	180 °C
Scan Begin	70 m/z	Set End Plate Offset	-500 V	Set Dry Gas	4.0 l/min
Scan End	1500 m/z	Set Charging Voltage	0 V	Set Divert Valve	Waste
		Set Corona	0 nA	Set APCI Heater	0 °C

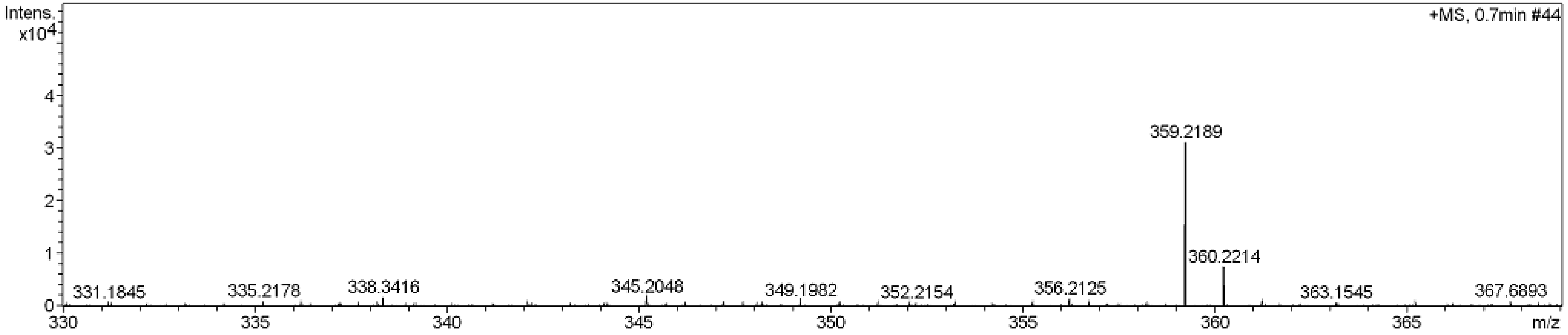

Meas. m/z	#	Ion Formula	Score	m/z	err [ppm]	err [mDa]	mSigma	rdb	e⁻ Conf	N-Rule
359.2189	1	C20H32NaO4	100.00	359.2193	-1.0	-0.4	12.5	4.5	even	ok
695.4492	1	C40H64NaO8	100.00	695.4493	-0.2	-0.1	51.6	8.5	even	ok

Figure S37. HR-ESI-MS spectrum of **3**.

Acquisition Parameter

Source Type	ESI	Ion Polarity	Positive	Set Nebulizer	0.4 Bar
Focus	Active	Set Capillary	4500 V	Set Dry Heater	180 °C
Scan Begin	70 m/z	Set End Plate Offset	-500 V	Set Dry Gas	4.0 l/min
Scan End	1500 m/z	Set Charging Voltage	0 V	Set Divert Valve	Waste
		Set Corona	0 nA	Set APCI Heater	0 °C

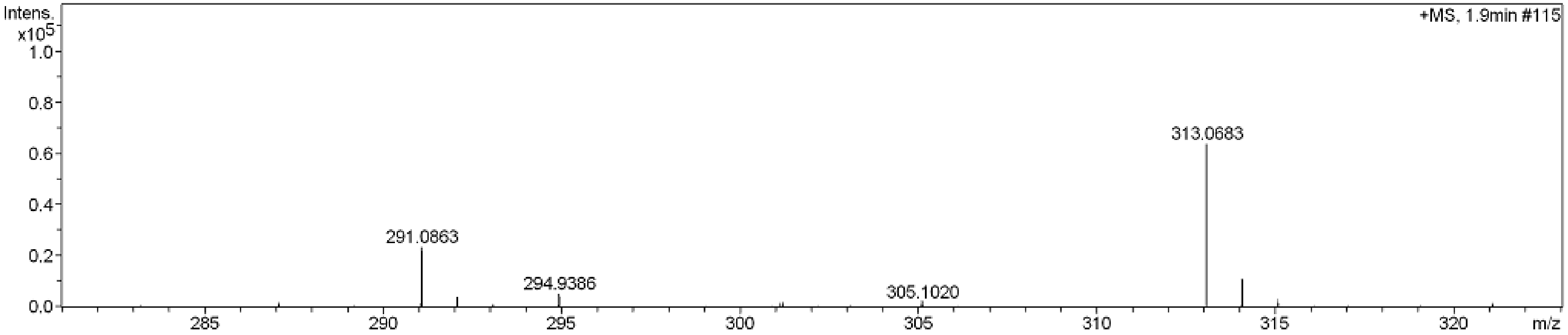

Meas. m/z	#	Ion Formula	Score	m/z	err [ppm]	err [mDa]	mSigma	rdb	e⁻ Conf	N-Rule
291.0863	1	$C_{15}H_{15}O_6$	100.00	291.0863	-0.2	-0.0	5.0	8.5	even	ok
313.0683	1	$C_{15}H_{14}NaO_6$	100.00	313.0683	-0.3	-0.1	5.5	8.5	even	ok
603.1469	1	$C_{30}H_{28}NaO_{12}$	100.00	603.1473	-0.7	-0.4	19.0	16.5	even	ok

Figure S38. HR-ESI-MS spectrum of **4**.

Acquisition Parameter

Source Type	ESI	Ion Polarity	Positive	Set Nebulizer	0.4 Bar
Focus	Active	Set Capillary	4500 V	Set Dry Heater	180 °C
Scan Begin	70 m/z	Set End Plate Offset	-500 V	Set Dry Gas	4.0 l/min
Scan End	1500 m/z	Set Charging Voltage	0 V	Set Divert Valve	Waste
		Set Corona	0 nA	Set APCI Heater	0 °C

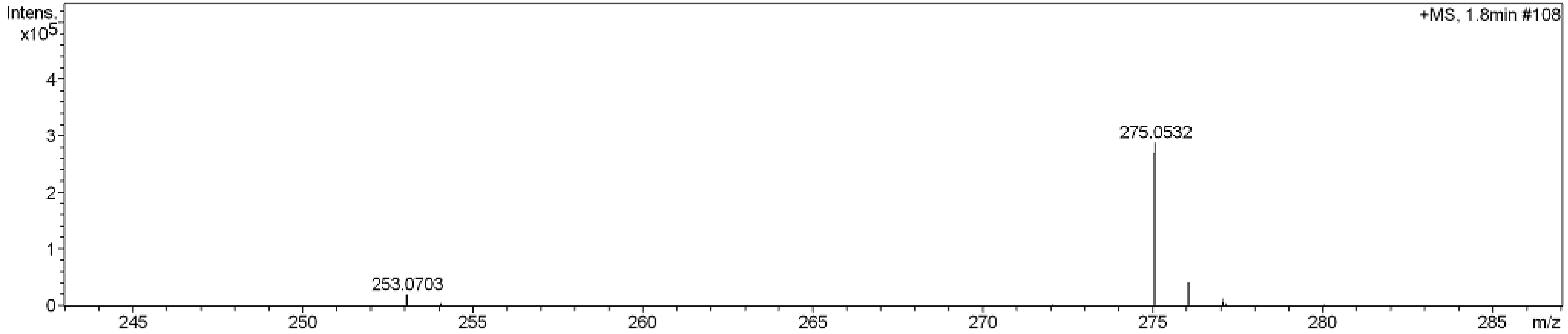

Meas. m/z	#	Ion Formula	Score	m/z	err [ppm]	err [mDa]	mSigma	rdb	e⁻ Conf	N-Rule
253.0703	1	C12H13O6	100.00	253.0707	1.3	0.3	5.2	6.5	even	ok
275.0532	1	C12H12NaO6	100.00	275.0526	2.0	0.6	6.7	6.5	even	ok
527.1159	1	C24H24NaO12	100.00	527.1160	-0.3	-0.1	2.8	12.5	even	ok

Figure S39. HR-ESI-MS spectrum of **6**.

Acquisition Parameter

Source Type	ESI	Ion Polarity	Positive	Set Nebulizer	0.4 Bar
Focus	Active	Set Capillary	4500 V	Set Dry Heater	180 °C
Scan Begin	100 m/z	Set End Plate Offset	-500 V	Set Dry Gas	4.0 l/min
Scan End	2000 m/z	Set Charging Voltage	0 V	Set Divert Valve	Waste
		Set Corona	0 nA	Set APCI Heater	0 °C

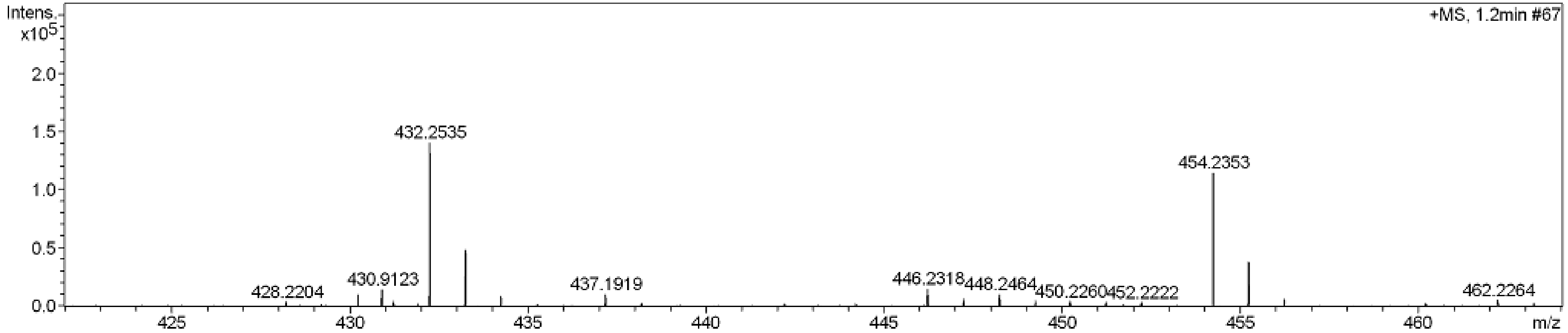

Meas. m/z	#	Ion Formula	Score	m/z	err [ppm]	err [mDa]	mSigma	rdb	e¯ Conf	N-Rule
432.2535	1	C28H34NO3	100.00	432.2533	0.5	0.2	18.6	12.5	even	ok
454.2353	1	C28H33NNaO3	100.00	454.2353	-0.1	-0.0	13.0	12.5	even	ok

Figure S40. HR-ESI-MS spectrum of **8**.

Acquisition Parameter

Source Type	ESI	Ion Polarity	Positive	Set Nebulizer	0.4 Bar
Focus	Active	Set Capillary	4500 V	Set Dry Heater	180 °C
Scan Begin	70 m/z	Set End Plate Offset	-500 V	Set Dry Gas	4.0 l/min
Scan End	1500 m/z	Set Charging Voltage	0 V	Set Divert Valve	Waste
		Set Corona	0 nA	Set APCI Heater	0 °C

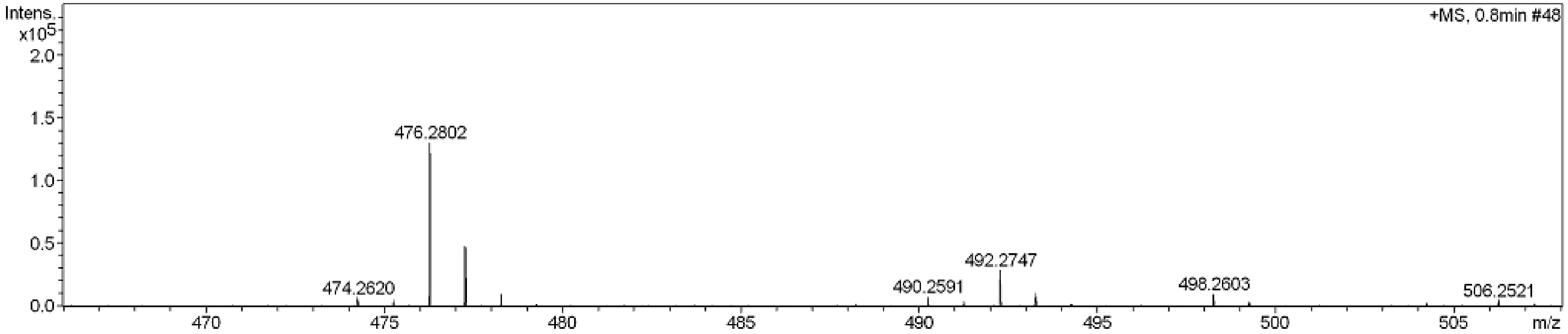

Meas. m/z	#	Ion Formula	Score	m/z	err [ppm]	err [mDa]	mSigma	rdb	e⁻ Conf	N-Rule
476.2802	1	C30H38NO4	100.00	476.2795	1.4	0.7	19.9	12.5	even	ok
498.2603	1	C30H37NNaO4	100.00	498.2615	2.3	1.1	14.4	12.5	even	ok
951.5530	1	C60H75N2O8	100.00	951.5518	1.3	1.2	29.9	24.5	even	ok
973.5319	1	C60H74N2NaO8	100.00	973.5337	-1.9	-1.8	47.5	24.5	even	ok

Table S1. The equation for Δε (E) calculations and energy analysis for the conformers of **1**.

$$\Delta\varepsilon(E) = \frac{1}{2.297 \times 10^{-39}} \cdot \frac{1}{\sigma\sqrt{\pi}} \sum_k E_{0k} R_{0k} \exp\left[-\left\{\frac{(E - E_{0k})}{\sigma}\right\}^2\right]$$

compounds	Conformation	G (Hartree)	G (Kcal/mol)	ΔG (Kcal/mol)	Boltzmann Dist (%)
**1**	**1a**	- 1387.70682701	-870790.0583	0	84.87%
	**1b**	-1387.70519965	-870789.0371	1.02	15.13%

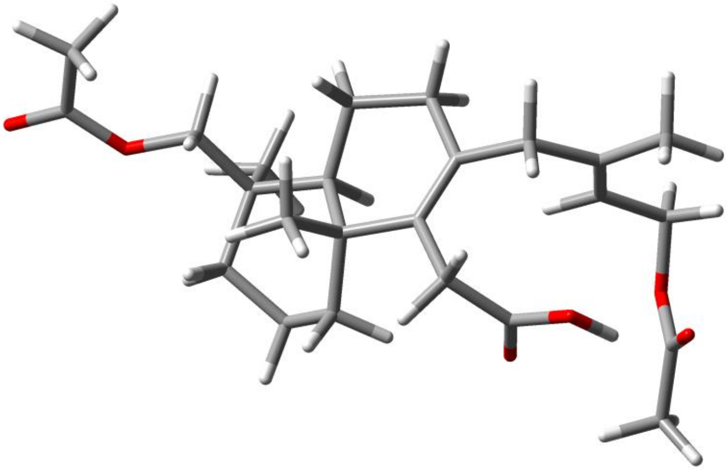

1a

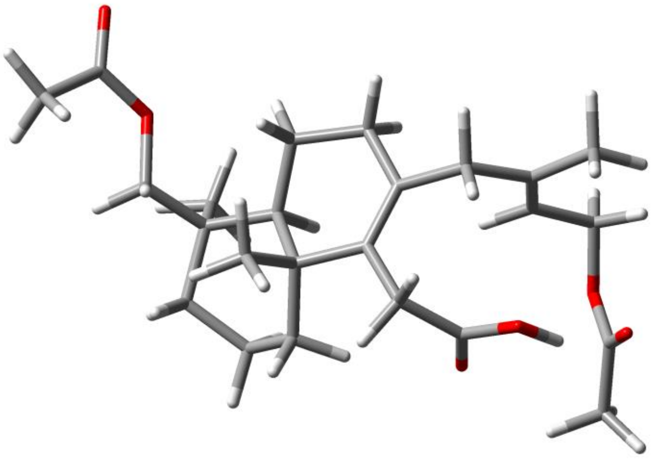

1b
